# Lysergic acid diethylamide (LSD) promotes social behavior through mTORC1 in the excitatory neurotransmission

**DOI:** 10.1073/pnas.2020705118

**Published:** 2021-01-25

**Authors:** Danilo De Gregorio, Jelena Popic, Justine P. Enns, Antonio Inserra, Agnieszka Skalecka, Athanasios Markopoulos, Luca Posa, Martha Lopez-Canul, He Qianzi, Christopher K. Lafferty, Jonathan P. Britt, Stefano Comai, Argel Aguilar-Valles, Nahum Sonenberg, Gabriella Gobbi

**Affiliations:** ^a^Neurobiological Psychiatry Unit, Department of Psychiatry, McGill University, Montreal, QC, Canada, H3A 1A1;; ^b^Department of Biochemistry, McGill University, Montreal, QC, Canada, H3A 1A3;; ^c^Department of Psychology, McGill University, Montreal, QC, Canada, H3A 1B1;; ^d^Division of Neuroscience, Vita Salute San Raffaele University, 20132 Milan, Italy;; ^e^Department of Neuroscience, Carleton University, Ottawa, ON, Canada, K1S 5B6;; ^f^McGill University Health Center, Montreal, QC, Canada, H3A 1A1

**Keywords:** LSD, social behavior, mTOR, 5-HT_2A_, AMPA

## Abstract

Clinical studies have reported that the psychedelic lysergic acid diethylamide (LSD) enhances empathy and social behavior (SB) in humans, but its mechanism of action remains elusive. Using a multidisciplinary approach including in vivo electrophysiology, optogenetics, behavioral paradigms, and molecular biology, the effects of LSD on SB and glutamatergic neurotransmission in the medial prefrontal cortex (mPFC) were studied in male mice. Acute LSD (30 μg/kg) injection failed to increase SB. However, repeated LSD (30 μg/kg, once a day, for 7 days) administration promotes SB, without eliciting antidepressant/anxiolytic-like effects. Optogenetic inhibition of mPFC excitatory neurons dramatically inhibits social interaction and nullifies the prosocial effect of LSD. LSD potentiates the α-amino-3-hydroxy-5-methyl-4-isoxazole propionate (AMPA) and 5-HT_2A_, but not *N*-methyl-D-aspartate (NMDA) and 5-HT_1A_, synaptic responses in the mPFC and increases the phosphorylation of the serine-threonine protein kinases Akt and mTOR. In conditional knockout mice lacking Raptor (one of the structural components of the mTORC1 complex) in excitatory glutamatergic neurons (*Raptor*^*f/f*^*:Camk2alpha-Cre*), the prosocial effects of LSD and the potentiation of 5-HT_2A_/AMPA synaptic responses were nullified, demonstrating that LSD requires the integrity of mTORC1 in excitatory neurons to promote SB. Conversely, in knockout mice lacking Raptor in GABAergic neurons of the mPFC (*Raptor*^*f/f*^*:Gad2-Cre*), LSD promotes SB. These results indicate that LSD selectively enhances SB by potentiating mPFC excitatory transmission through 5-HT_2A_/AMPA receptors and mTOR signaling. The activation of 5-HT_2A_/AMPA/mTORC1 in the mPFC by psychedelic drugs should be explored for the treatment of mental diseases with SB impairments such as autism spectrum disorder and social anxiety disorder.

## Significance

Social behavior (SB) is a fundamental hallmark of human interaction. Repeated administration of low doses of the 5-HT_2A_ agonist lysergic acid diethylamide (LSD) in mice enhances SB by potentiating 5-HT_2A_ and AMPA receptor neurotransmission in the mPFC via an increasing phosphorylation of the mTORC1, a protein involved in the modulation of SB. Moreover, the inactivation of mPFC glutamate neurotransmission impairs SB and nullifies the prosocial effects of LSD. Finally, LSD requires the integrity of mTORC1 in excitatory glutamatergic, but not in inhibitory neurons, to produce prosocial effects. This study unveils a mechanism contributing to the role of 5-HT_2A_ agonism in the modulation of SB.

Social behavior (SB) is defined as a set of complex interactions among individuals, normally within the same species, that are usually beneficial to one individual or group. The prefrontal cortex (PFC) exerts a pivotal role in the modulation of SB in mammals (1). PFC pathology engenders impaired SB (2) Disrupted SB is prevalent in various mental illnesses, including autism spectrum disorder (ASD) and social anxiety disorders (SAD) (3), for which few definitive therapies are available.

In the past decade, psychedelic drugs have been investigated as potential therapeutics in psychiatry. For example, the acute administration of lysergic acid diethylamide (LSD) in humans produces feelings of happiness, trust, closeness to others, and enhanced emotional empathy (4), while promoting positive mood changes, altruistic/positive social effects, and well-being/life satisfaction (5). Despite these positive results, the mechanism of action underlying the prosocial effects of LSD remains elusive. LSD is a partial agonist of the serotonin 5-HT_2A_ receptor (6), but also displays affinity for the 5-HT_1A_ receptor (7–9). Several studies have demonstrated that LSD modulates glutamatergic neurotransmission and, indirectly, the α-amino-3-hydroxy-5-methyl-4-isoxazole propionate (AMPA) receptors. Indeed, in vitro studies have shown that LSD increased the excitatory response of interneurons in the piriform cortex following AMPA (5 µM) application (10). Moreover, the 5-HT_2A_ receptor stimulation produces excitatory postsynaptic potentials (EPSPs) in layer V of the medial PFC (mPFC), thus promoting a release of glutamate in the apical dendritic region of layer V pyramidal cells in the mPFC, which in turn activates the AMPA receptors (11). Intriguingly, activation of the AMPA receptor promotes the activation of the protein serine/threonine kinase mammalian/mechanistic target of rapamycin complex 1 (mTORC1) signaling, which is dysfunctional in several diseases displaying impairment in SB (12). Moreover, recent evidence showed that the sensitization of the 5-HT_2A_ receptor requires the recruitment of the mTORC1 pathway (13). In addition, an in vitro study showed that LSD and other psychedelics increase spine density in cortical neurons, an effect reverted by the mTORC1 inhibitor rapamycin (14). However, the role of 5-HT_2A_ and AMPA receptors as well as that of mTORC1 in orchestrating the behavioral effects of LSD in SB has not yet been investigated. Here, we dissected the neurobiological mechanisms underlying the prosocial effects of LSD in mice. Employing a multidisciplinary approach, we found that repeated, but not acute, administration of LSD promotes SB by activating cortical AMPA and 5-HT_2A_ responses, and that these effects require the integrity of excitatory transmission in the mPFC and an intact mTORC1 complex in excitatory, but not inhibitory neurotransmission.

## Results

### Repeated LSD Treatment Increases Social Interaction in the Direct Social Interaction Test (DSI) and the Three Chambers Test (TCT).

**Fig. 1.**
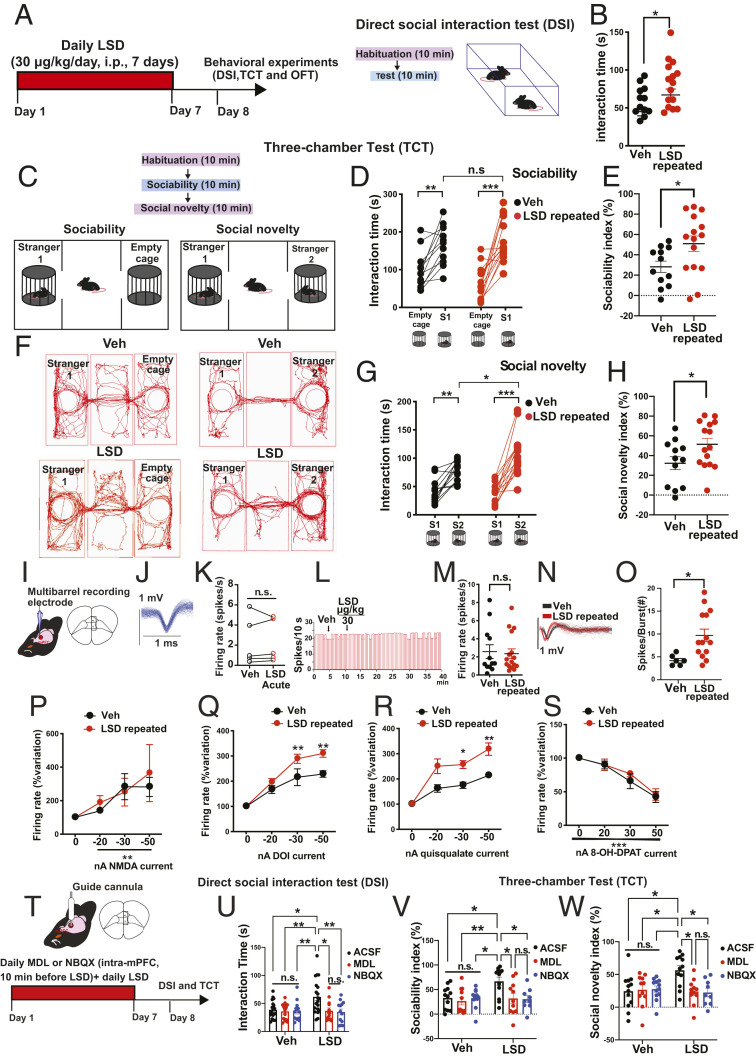
Repeated LSD treatment exerts prosocial effects and potentiates the synaptic response to 5-HT_2A_ and AMPA receptor agonists, but not NMDA and 5-HT_1A_ receptor agonists in the mPFC. (*A*) Scheme illustrating the timing of treatment and behavioral experiments. (*B*) Mice treated with LSD (30 μg/kg/day, i.p., for 7 d) spent more time with a stranger mouse in the DSI (*n* = 12 to 15 mice per group). Each line represents mean ± SEM and each point represents a single mouse. Student’s unpaired two-tailed *t* test. (*C*) Scheme representing the TCT. (*D*) Interaction time spent with the unfamiliar mouse (S1) versus the empty cage in mice treated with Veh or LSD (*n* = 12 to 15 mice per group). One-way ANOVA unmatched followed by Bonferroni post hoc analysis. (*E*) Summary of sociability index. Student’s unpaired two-tailed *t* test. (*F*) Representative exploratory activity of mice treated with Veh (*Top*) or LSD (*Bottom*) in the three-chamber test. (*G*) Time spent with the familiar mouse (S1) versus the unfamiliar mouse (S2) in mice treated with Veh or LSD (*n* = 12 to 15 mice per group). One-way ANOVA unmatched followed by Bonferroni post hoc analysis. (*H*) Summary of social novelty index. Each bar represents mean ± SEM and each point represents the result of each mouse tested in each group; one-way ANOVA followed by Bonferroni post hoc comparisons. Student’s unpaired two-tailed *t* test. (*I*) Representation of coronal sections of the mouse brain of the recording site in the mPFC. Prelimbic (PL); infralimbic (IL). (*J*) Typical spike waveform of a glutamatergic pyramidal neuron in the mPFC. (*K*) Acute administration of LSD (30 μg/kg, i.p.) did not modify the basal firing activity (*n* = 5 mice), one neuron per animal. The acute injection of Veh did not change the firing activity (Student’s unpaired two-tailed *t* test). (*L*) Representative firing rate histograms of a pyramidal neuron. Black arrows indicate the injection of Veh and LSD. (*M*) Repeated LSD (30 μg/kg/day, i.p., for 7 d) did not change the firing rate activity. Each line represents mean ± SEM and each point represents a single neuron recorded in each group (Student’s unpaired two-tailed *t* test). (*N*) Representative overdraw waveform of pyramidal neurons of mice treated with Veh (black) or repeated LSD (red). (*O*) Repeated LSD increased the number of spikes per burst. LSD did not change the excitatory response of increasing currents of NMDA (*P*) but potentiates the excitatory response of the selective 5-HT_2A_ DOI (*Q*) and the excitatory response upon the ejection of the selective (*R*) AMPA agonist quisqualate. (*S*) No significant difference was observed between repeated LSD and Veh in the inhibitory response of increasing current following the ejection of the selective 5-HT_1A_ agonist 8-OH-DPAT. Two-way ANOVA for repeated measures (RM) followed by Bonferroni post hoc comparisons. (*T*) Representation of the implanted cannula in the mPFC (PL and IL regions, *Top*) and of the scheme illustrating the timing of treatment and behavioral experiments (*Bottom*). (*U*) Repeated intra-mPFC infusions of the selective 5-HT_2A_ antagonists MDL 100 907 or the selective AMPA antagonists NBQX blocked the increase in interaction time induced by LSD (*n* = 13 to 20 mice). (*V*) Summary of sociability index showing that MDL and NBQX blocked the prosocial effect of LSD. (*W*) Summary of social novelty index showing that MDL and NBQX blocked the ability of LSD to increase the preference for social novelty (*n* = 9 to 12 mice). One-way ANOVA or two-way ANOVA followed by Bonferroni post hoc analysis. Each bar represents mean ± SEM and each point represents a single mouse tested in each group. *P < 0.05, **P < 0.01, ***P < 0.001; n.s., not significant. ACSF, artificial cerebrospinal fluid. Detailed data and post hoc analysis are available in *SI Appendix*, Table S2.

We first investigated whether a single (30 μg/kg, i.p.) or repeated (30 μg/kg/day, i.p., for 7 d) LSD administration has an effect on SB. Mice treated with repeated (Fig. 1 *A* and *B*) but not a single (*SI Appendix*, Fig. S1) dose of LSD spent more time interacting with a stranger mouse in the direct social interaction test (DSI), compared with mice treated with vehicle (Veh). We were also interested to see if this repeated regimen had an effect on despair, anhedonia, anxiety, and stereotypic behavior. However, repeated administration of LSD in the novelty suppressed feeding test (NSFT) did not induce any change in the latency to feed in the new environment (*SI Appendix*, Fig. S3*A*) or in the home cage (*SI Appendix*, Fig. S3*B*). Moreover, LSD did not reduce the immobility time in the forced swim test (FST) (*SI Appendix*, Fig. S4*A*) and did not alter the sucrose preference percentage (*SI Appendix*, Fig. S4*B*), the total amount of sucrose (*SI Appendix*, Fig. S4*C*), and the total amount of volume (sucrose and water) consumed (*SI Appendix*, Fig. S4*D*) in the sucrose preference test (SPT), thus ruling out antidepressant or prohedonic effects at our chosen dose. To confirm that the prosocial effect is not mouse specific, we tested male rats with repeated LSD doses, and consistently observed an increased interaction time with a stranger rat in the DSI, compared to Veh (*SI Appendix*, Fig. S2).

After the DSI, the same cohort of mice underwent the TCT. In the first phase of the test, an adult mouse normally spends more time in a chamber containing a stranger mouse (S1, intruder mouse) than in an empty chamber (this is an index of sociability). In the second phase, the same test mouse will spend more time investigating another unfamiliar stranger (S2, novel intruder mouse) than the familiar conspecific (this is an index of preference for social novelty). We quantified the time spent with S1 and empty cage using the “sociability index” (15), and time spent with S1 and S2 using the “social novelty index” (*SI Appendix*). When given a choice, both mice treated with Veh or LSD showed an increased interaction time with a stranger (S1) mouse over the empty cage, but mice treated with LSD showed a decreased preference score for the empty cage during the sociability phase (*SI Appendix*, Fig. S5*A*). Remarkably, the sociability index (Fig. 1*D*) was higher in the group treated with LSD compared with Veh. Morover, when mice treated with Veh or LSD were directly exposed to a novel mouse (S2), the LSD-treated group displayed an increased interaction time toward the stranger mouse compared to Veh (Fig. 1 *G* and *H*), thus demonstrating that LSD engenders a stronger preference also for social novelty. LSD did not modify the absolute number of contacts with S1 (*SI Appendix*, Fig. S5*B*) and the S2 mouse (*SI Appendix*, Fig. S5*C*). No statistical difference was detected in the distance traveled when mice were allowed to explore the three-chamber apparatus during the habituation (*SI Appendix*, Fig. S5*D*), the sociability (*SI Appendix*, Fig. S5*E*), or the social novelty phase (*SI Appendix*, Fig. S5*F*). No effect of LSD was detected on the time spent in each chamber during both social preference and social novelty phases (*SI Appendix*, Fig. S5 *A* and *B*). The effects on locomotion were further investigated in the open field test (OFT) and we found no changes in the distance traveled (*SI Appendix*, Fig. S6*A*) and no difference in the time spent in the center (*SI Appendix*, Figs. S6 *B* and *D*) or number of entries in the center (*SI Appendix*, Fig. S6 *C* and *D*) of the arena, further confirming the lack of anxiolytic properties of LSD at our chosen dose. During the OFT, we also assessed stereotypic behavior. No differences were detected in the duration and in the number of grooming episodes (*SI Appendix*, Fig. S6 *E* and *F*), nor in the duration (*SI Appendix*, Fig. S6*G*) and the number of rearing episodes (*SI Appendix*, Fig. S6*H*) between mice treated with LSD or Veh. These results demonstrate that LSD, at this regimen, selectively increases social interaction and preference for social novelty without affecting locomotion and stereotypic behavior or anxiety- and depressive-like behaviors.

### Repeated LSD Treatment Enhances Microiontophoretic Responses of 5-HT_2A_ and AMPA, but Not 5-HT_1A_ and *N*-Methyl-d-Aspartate (NMDA) Receptors on mPFC Pyramidal Neurons.

Next, we investigated which receptors mediate the prosocial effects of LSD within the mPFC. Brain imaging studies in humans have shown that the effects of LSD on SB are mediated by mPFC 5-HT_2A_ receptors (16, 17), and that the activation of postsynaptic cortical 5-HT_2A_ receptors increases glutamate release in the synaptic cleft, an effect that can be reversed by AMPA antagonists like LY293558 (11, 18). Therefore, employing in vivo electrophysiological recordings with multibarreled electrodes for microiontophoretic injections (19), we investigated the ability of LSD to modulate firing and burst activity of pyramidal neurons in both prelimbic (PL) and infralimbic (IL) regions of the mPFC (Fig. 1 *I* and *J*). Moreover, we assessed the excitatory response to microiontophoretic ejections of the 5-HT_2A_ selective agonist (±)-2,5-dimethoxy-4-iodoamphetamine (DOI) (19) and of the AMPA selective agonist quisqualate (excitatory responses in mice treated with Veh or LSD) (20). The basal firing activity of pyramidal neurons was unchanged after a single (Fig. 1 *K* and *L*) and repeated (Fig. 1 *M* and *N*) LSD administration. In contrast, repeated LSD increased the burst activity of mPFC pyramidal neurons (as measured by the number of spikes per burst in 200 s) (Fig. 1*O*). Moreover, repeated LSD administration potentiated, in a current-dependent manner, the excitatory response to DOI (Fig. 1*Q*), and the excitatory response upon the ejection of quisqualate (Fig. 1*R*), but did not affect the excitatory responses of increasing excitatory NMDA currents (20) (Fig. 1*P*). No significant differences were found between repeated LSD and Veh in the inhibitory response of increasing current following the ejection of the selective 5-HT_1A_ agonist 8-hydroxy-2-(DI-n-propylamino)tetralin hydrobromide (8-OH-DPAT) (19) (Fig. 1*S*). These data indicate that repeated LSD selectively potentiates AMPA and 5-HT_2A_ excitatory transmission of the mPFC.

### 5-HT_2A_ and AMPA Receptors Mediate the Prosocial Effects of LSD in the DSI and TCT.

Next, we investigated the role of cortical 5-HT_2A_ and AMPA receptors on SB in mice repeatedly treated with LSD. The selective 5-HT_2A_ receptor antagonist (R)-(+)-α-(2,3-dimethoxyphenyl)-1-[2-(4-fluorophenyl) ethyl]-4-piperinemethanol [MDL 100 907 (MDL)] and the selective AMPA receptor antagonist 2,3-dihydroxy-6-nitro-7-sulfamoyl-benzo[f]quinoxaline-2,3-dione (NBQX), or the artificial cerebrospinal fluid (ACSF) were microinfused for 7 d into the mPFC, 10 min before the daily systemic injection of LSD (Fig. 1*T*). Pretreatment with MDL and NBQX nullified the prosocial effect of LSD in the DSI (Fig. 1*U*). Similarly, mPFC injection of MDL and NBQX blocked the LSD-induced increase of the sociability index in the TCT (Fig. 1*V*). In the second phase of the test, when mice were directly exposed to a novel mouse, the social novelty index of animals pretreated with MDL or NBQX and LSD was not different from animals treated with Veh, demonstrating that the two receptor antagonists blocked the increased preference for social novelty induced by LSD (Fig. 1*W*). MDL and NBQX when microinfused alone in mice treated with Veh (intraperitoneally [i.p.]), did not affect the interaction time in the DSI and TCT, (Fig. 1 *U*, *V*, and *Q*), thus ruling out any effect of the antagonists on sociability, per se. These experiments show that the prosocial effects induced by LSD require neurotransmission through mPFC 5-HT_2A_ and AMPA receptors.

### Optogenetic Photoinhibition of Excitatory Neurons in the mPFC Induces Social Avoidance and Blocks the Prosocial Effects of LSD.

**Fig. 2.**
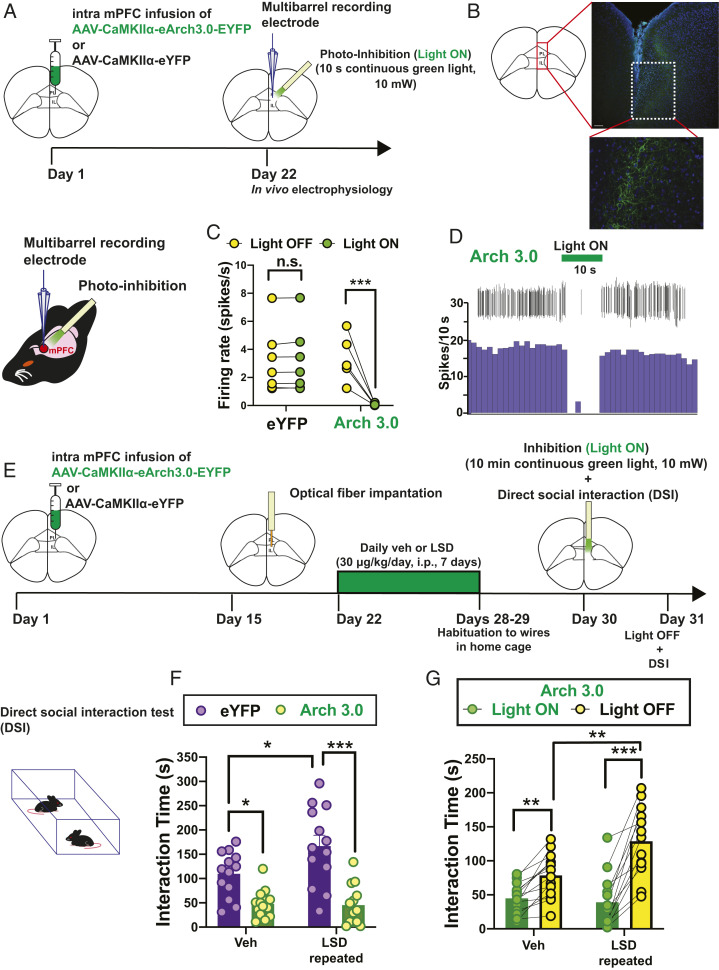
Optogenetic photoinhibition of excitatory neurons in the mPFC nullifies the prosocial effects of repeated LSD. (*A*) Timeline showing the infusion of AAV-CaMKIIα-eArch3.0-eYFP or the control AAV-CaMKIIα-eYFP in the mPFC. Twenty-two days after the viral injection, C57BL/6J mice were prepared for in vivo electrophysiological recordings of mPFC-pyramidal neurons. An optical fiber was lowered into the mPFC with an angle of 20°. (*B*) Green fluorescent protein showing the expression of AAV-CaMKIIα-eArch3.0-eYFP in the mPFC. (*C*) Green light illumination (10 mW, 532 nm, 10 s on) produced a decrease of the firing activity of pyramidal-mPFC neurons in animals infused with AAV-CaMKIIa-eArch3.0-eYFP (five neurons recorded in two mice) but not AAV-CaMKIIα-eYFP (six neurons recorded in two mice). RM two-way ANOVA followed by Bonferroni post hoc comparisons. (*D*) Firing rate histogram of a single pyramidal mPFC neuron showing a decrease during laser illumination (10 mW, 532 nm, 10 s on). (*E*) Timeline showing the infusion of AAV-CaMKIIα-eArch3.0-eYFP or the control AAV-CaMKIIα-eYFP in the mPFC. Fifteen days after the viral injection, each animal was implanted with an optical fiber. Seven days postsurgery, animals were treated with Veh or LSD (30 μg/kg/day, i.p., for 7 d) and tested 24 h after the last injection in the DSI. Animals received laser photoinhibition during the whole duration of the test (10 mW, 532 nm, for 10 min). (*F*) Photoinhibition of mPFC excitatory neurons decreased the interaction time in animals receiving AAV-CaMKIIα-eArch3.0-eYFP. Importantly, LSD failed to elicit prosocial effects in animals that had received the infusion of AAV-CaMKIIα-eArch3.0-eYFP, but not in animals that had received the control AAV-CaMKIIα-eYFP. Two-way ANOVA followed by Bonferroni post hoc comparisons (*G*). Twenty-four hours later, mice infused with AAV-CaMKIIα-eArch3.0-eYFP were tested with the laser light turned off. (*n* = 13 to 14 mice). RM two-way ANOVA followed by Bonferroni post hoc comparisons. *P < 0.05, **P < 0.01, ***P < 0.001; n.s., not significant. Detailed data and post hoc analyses are available in *SI Appendix*, Table S2.

The PFC is a brain region strongly involved in SB (1, 21, 22). We sought to determine whether mPFC photoinhibition affects social interaction in mice, and whether mPFC integrity is required for LSD to exert its prosocial effects. To silence cortical excitatory neurons, we injected an adeno-associated virus vector expressing archaerhodopsin 3.0 (Arch 3.0) under the control of the calcium/calmodulin-dependent protein kinase II α (CaMKIIα) promoter (AAV-CamKIIα-Arch.3.0-eYFP) into the mPFC. First, following the expression of AAV-CamKIIα-Arch.3.0-eYFP in the mPFC (Fig. 2 *A* and *B*), 10 s of 530 nm light delivered to the mPFC completely shut down the firing activity of mPFC pyramidal neurons, but not in animals infused with the control virus AAV-CamKIIα-eYFP (Fig. 2 *C* and *D*). The effects of photoinhibition were then assessed in the DSI paradigm. We tested whether LSD requires the integrity of mPFC excitatory neurons to exert its prosocial effects (Fig. 2*E*). Following photoinhibition of mPFC glutamatergic neurons (10 min of continuous green light), mice that were infused with AAV-CamKIIα-Arch.3.0-eYFP and treated with repeated LSD (last dose 24 h before the light-on experiments) displayed decreased interaction time toward the unfamiliar conspecific mouse in the DSI, similar to the mice receiving Veh. In contrast, mice with control AAV-CamKIIα-eYFP infusion, treated with LSD, displayed increased social interaction compared to mice treated with Veh when the laser light was on (Fig. 2*F*). In addition, the same cohort of mice treated with Veh or LSD, which received an AAV-CamKIIα-Arch.3.0-eYFP infusion, were tested again 24 h later with the laser turned off. When the light was turned off the next day, LSD-treated mice (last dose 48 h before the light-off experiments) displayed increased interaction time toward the stranger mouse, suggesting that photoinhibition of mPFC glutamatergic neurons transiently blocks the prosocial effects of LSD. Interestingly, the interaction time was still higher in LSD-treated mice when compared to Veh-treated mice, suggesting that the prosocial effects of LSD last more than 48 h after the last injection (Fig. 2*G*). These results suggest that the prosocial effects of LSD require intact and functioning mPFC excitatory neurotransmission.

### Repeated LSD Treatment Increases Akt and mTOR Phosphorylation in the PFC.

**Fig. 3.**
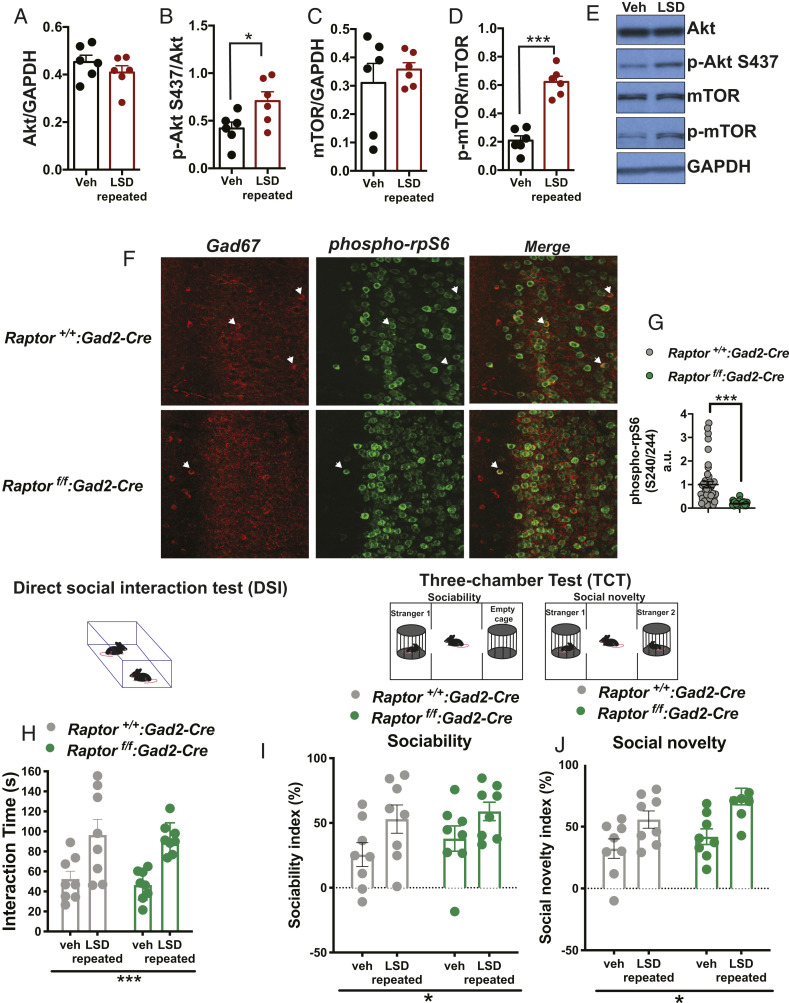
Intact mTORC1 complex in inhibitory GABAergic neurotransmission is not necessary for the prosocial effects of LSD. (*A*) Repeated LSD (30 μg/kg/day, i.p., for 7 d) did not change total Akt levels in the PFC but (*B*) significantly increased Akt S437 phosphorylation compared with Veh (*n* = 6 mice per group). (*C*) Repeated LSD did not change total mTOR levels in the PFC but (*D*) significantly increased mTOR phosphorylation compared with Veh (*n* = 6 mice per group). (*E*) Representative Western blots of Akt, p-Akt S437, mTOR, and p-mTOR quantification for mice treated with Veh or LSD. Each bar represents mean ± SEM and each point represents a single mouse value. Student’s unpaired two-tailed *t* test. (*F*) Raptor, a defining element of mTORC1, was knocked out from GAD67-positive neurons (*Raptor*^*f/f*^*:Gad2-Cre*). Representative immunohistochemistry images demonstrating the effect of Raptor’s absence on phosphorylation of the ribosomal protein S6 (rpS6; ser240/244) in GAD67-positive neurons of the medial prefrontal cortex. White arrows indicate GAD67-positive neurons colocalizing with phospho-rpS6. (*G*) Quantification of the phospho-rpS6 in GAD67-positive neurons in control (*Raptor*^*+/+*^*:Gad2-Cre*) and *Raptor*^*f/f*^*/Gad2-Cre* mice. Each point represents a single GAD67-positive neuron colocalizing with phospho-rpS6. Unpaired two-tailed *t* test with Welch’s correction. (*H*) Repeated LSD (30 μg/kg/day, i.p., for 7 d) increased direct social interaction with an unfamiliar mouse in both *Raptor*^*+/+*^*:Gad2-Cr*e and *Raptor*^*f/f*^*/Gad2-Cre* mice compared to Veh (*n* = 8 mice per group). (*I*) Summary of sociability index showing that LSD increased sociability in both control littermates and *Raptor*^*f/f*^*:Gad2-Cre* mice. (*J*) Summary of social novelty index showing that LSD increased preference for social novelty in both wild-type and *Raptor*^*f/f*^*:Gad2-Cre* mice (*n* = 8 mice per group). Two-way ANOVA analysis. *P < 0.05, ***P < 0.001. Detailed data are available in *SI Appendix*, table S2.

The mTOR pathway is activated by several psychoactive drugs, including the hallucinogen ketamine (23, 24) and selective serotonin reuptake inhibitor (SSRI) antidepressants (25). Furthermore, dysregulation of mTORC1 signaling is implicated in social deficit disorders (12, 26, 27). The mTORC1 complex can be activated via AMPA receptors (24) and through the phosphatidylinositol 3-kinase (PI3K) and serine/threonine kinase B (Akt) pathways (12, 28). Thus, we measured the level of the serine-threonine protein kinase Akt S437 and mTOR phosphorylation after repeated administration of LSD. Western blot analysis revealed that LSD had no effect on total Akt (Fig. 3*A*) and mTOR (Fig. 3*C*) levels, but significantly increased the phosphorylation levels of the proteins (Fig. 3 *B* and *D*, respectively) in the PFC, demonstrating that mTOR signaling is activated by repeated LSD administration in the mPFC.

### Intact mTORC1 Complex in Inhibitory Transmission Is Not Essential for the Prosocial Effects of LSD.

To further corroborate the role of mTOR, and, specifically, of mTORC1 in the prosocial effects elicited by LSD, we generated conditional knockout mice lacking *Raptor*, a subunit of the mTORC1 complex, in both excitatory (*Raptor*^*f/f*^*/Camk2α-Cre*) and inhibitory (*Raptor*^*f/f*^*/Gad2-Cre*) neurons. *Raptor* is an adaptor protein that recruits mTOR substrates, including the ribosomal protein S6 (rpS6) kinase which phosphorylates rpS6 (phospho-rpS6; ser240/244) (29, 30). First, to explore the role of mTORC1 in inhibitory neurons in mediating the behavioral effects of LSD, we performed experiments in mice lacking *Raptor* in *Gad2-*positive neurons (31), which are GABAergic inhibitory neurons. Immunohistochemical analysis showed a decreased amount of phospho-rpS6 protein in *Raptor*^*f/f*^*/Gad2-Cre* mice compared to control mice (*Raptor*^*+/+*^*/Gad2-Cre*), specifically in inhibitory neurons (Gad67^+^ cells, Fig. 3 *F* and *G*). LSD increased the interaction time in the DSI (Fig. 3*H*) and promoted sociability (Fig. 3*I*) and preference for social novelty (Fig. 3*J*) in the TCT similarly in both control (*Raptor*^*+/+*^*/Gad2-Cre*) and *Raptor*^*f/f*^*/Gad2-Cre* mice. Together, these results rule out the direct involvement of the mTORC1 complex in inhibitory neurons on LSD-induced prosocial behavior.

### Intact mTORC1 Complex in Excitatory Transmission Is Required for the Prosocial Effects of LSD.

**Fig. 4.**
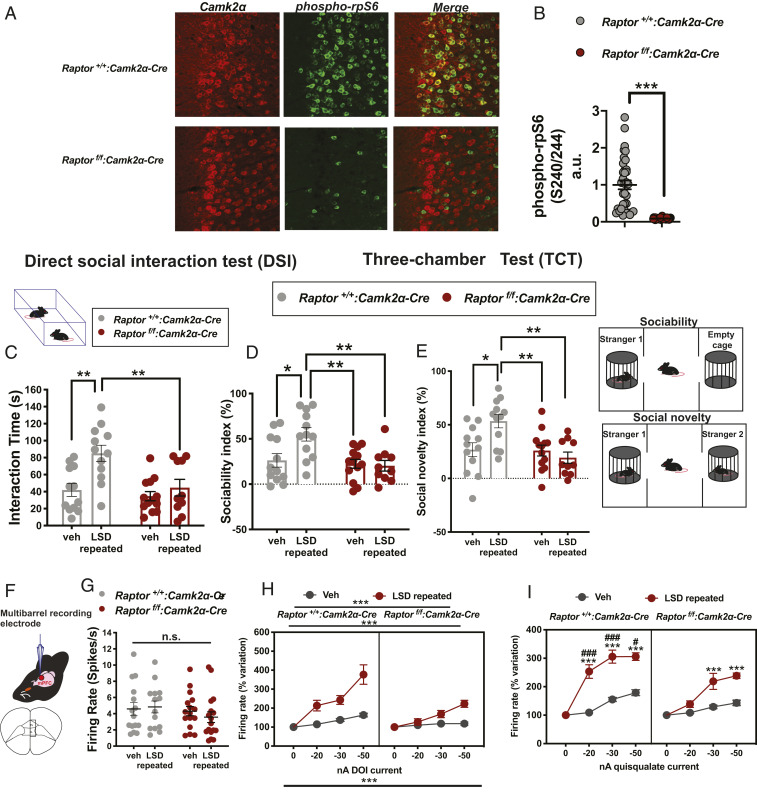
Intact mTORC1 complex in excitatory transmission is necessary for the prosocial effects of LSD and for the potentiation of 5-HT_2A_ and AMPA receptors induced by LSD. (*A*) Raptor, a constitutive element of mTORC1, was knocked out from Camk2α-positive neurons (*Raptor*^*f/f*^*:Camk2α-Cre*). Representative immunohistochemistry images demonstrating the effect of Raptor’s absence on phosphorylation of the ribosomal protein S6 (rpS6; ser240/244) in the medial prefrontal cortex. (*B*) Quantification of the phospho-rpS6 in Camk2α-positive neurons in the controls (*Raptor*^*+/+*^*:Camk2α-Cre*) and *Raptor*^*f/f*^*:Camk2α-Cre* mice. Each point represents a single Camk2α-positive neuron colocalizing with phospho-rpS6. Unpaired two-tailed *t* test with Welch’s correction. (*C*) Repeated LSD administration significantly increased the interaction time in the DSI in control littermates (*Raptor*^*+/+*^*:Camk2α-Cre*) but not in mutant *Raptor*^*f/f*^*:Camk2α-Cre* mice. (*D*) Summary of sociability index showing that LSD increased sociability in the control but not in mutant *Raptor*^*f/f*^*:Camk2α-Cre* mice. (*E*) Summary of social novelty index showing that LSD induced preference for social novelty in the control but not in mutant *Raptor*^*f/f*^*:Camk2α-Cre* mice (*n* = 10 to 13 mice per group). Two-way ANOVA analysis followed by Bonferroni post hoc comparisons. (*F*) Representation of coronal sections of the mouse brain in the mPFC. Prelimbic (PL); infralimbic (IL). (*G*) No significant differences were found in the firing activity of the pyramidal neurons in mutant and control mice treated with repeated Veh or LSD. Two-way ANOVA analysis. (*H*) Repeated LSD potentiated the excitation induced by 5-HT_2A_ agonists DOI ejections in both wild-type and mutant mice, even to a lesser extent in *Raptor*^*f/f*^*:Camk2α-Cre* mice. (*I*) LSD increased the excitation induced by quisqualate agonist current ejections in both genotypes, even to a lesser extent in mutant *Raptor* ^*f/f*^*:Camk2α-Cre* mice (*n* = 14 to 17 neurons per group). Three-way ANOVA followed by Bonferroni post hoc comparison. Each point of the line represents mean ± SEM expressed as percentage of baseline increasing current application. Two-way ANOVA followed by Bonferroni post hoc comparisons. *P < 0.05, **P < 0.01, ***P < 0.001 vs. Veh. ^#^*P* < 0.05, ^###^*P* < 0.001 vs. *Raptor*^*f/f*^*:Camk2α-Cre*. n.s., not significant. Detailed data and post hoc analysis are available in *SI Appendix*, Table S2.

To explore the role that mTORC1 in excitatory neurons plays in mediating the behavioral effects of LSD, we performed experiments in mice lacking Raptor in *Camk2α*-positive neurons, a marker used to target the neurotransmission (32). The absence of phospho-rpS6 protein in *Raptor*^*f/f*^*/Camk2α-Cre* mice, compared to control littermates (*Raptor*^*+/+*^*/Camk2α-Cre*) (Fig. 4 *A* and *B*) confirmed the knockout of *Raptor* in excitatory *Camk2α-Cre*-positive neurons in the PFC in *Raptor*^*f/f*^*/Camk2α-Cre* mice. Remarkably, treatment with LSD increased the interaction time in control, but not in *Raptor*^*f/f*^*/Camk2α-Cre* mice in the DSI paradigm (Fig. 4*C*). LSD also increased the sociability index in control (*Rapto*r^*+/+*^*/Camk2α-Cre*), but not in the mutant *Raptor*^*f/f*^*/Camk2α-Cre* line (Fig. 4*D*). Similarly, in the mutant *Raptor*^*f/f*^*/Camk2α-Cre* mice, unlike in control mice, LSD did not increase the social novelty index (Fig. 4*E*). Next, we determined whether the abnormal behavioral response in *Raptor*^*f/f*^*/Camk2α-Cre* was coupled with a perturbed response of 5-HT_2A_ and AMPA-mediated excitation in the mPFC. Using in vivo electrophysiology and microiontophoresis, LSD elicited an increase in evoked firing activity following the application of DOI (Fig. 4*H*) and quisqualate (Fig. 4*I*) in both *Raptor*^*f/f*^*/Camk2α-Cre* and control littermates, but this effect was significantly attenuated in *Raptor*^*f/f*^*/Camk2α-Cre* (Fig. 4 *H* and *I*). Three-way ANOVA showed a significant difference for both DOI and quisqualate responses between *Raptor*^*f/f*^*/Camk2α-Cre* and their littermates treated with Veh, demonstrating that the lack of *Raptor* in excitatory neurons produces an imbalanced 5-HT_2A_/AMPA excitatory response in the mPFC. Overall, these results show that the LSD effects are mediated by the mTORC1 complex in excitatory neurons to promote social interaction and to activate mPFC pyramidal neurons through 5-HT_2A_ and AMPA receptors.

## Discussion

This study identifies a mechanism contributing to the prosocial effects of the psychedelic compound LSD. Repeated, but not single LSD administration increases SB in male mice. This effect is mediated by the potentiation of 5-HT_2A_ and AMPA neurotransmission in the mPFC. Importantly, we demonstrated that LSD activates the serine-threonine protein kinase Akt and mTOR and that mTORC1 is essential for the effect of LSD on SB, through the 5-HT_2A_ and AMPA receptors. Specifically, the mTORC1 complex in mPFC glutamatergic excitatory neurons is necessary to elicit the prosocial effects of LSD as well as the LSD-evoked 5-HT_2A_ and AMPA responses. The importance of mPFC glutamatergic transmission in SB was also confirmed by the optogenetic inhibition of excitatory neurons, since this treatment completely nullifies the behavioral outcomes produced by LSD.

The mPFC has been extensively studied for its role in SB, as patients with mPFC lesions show increased social isolation and apathy (1). Layer V pyramidal glutamatergic neurons are the main output of the mPFC, and it is through these neurons that LSD exerts its behavioral effects, as observed in previous pharmacological and brain imaging studies (33, 34). We found that optogenetic inhibition of the mPFC excitatory neurons produced social avoidance in mice during the DSI, further supporting the notion that the integrity of mPFC pyramidal neurons is necessary for SB. Moreover, LSD failed to increase sociability in mice during the optically induced silencing of cortical excitatory transmission, thus bolstering the hypothesis that LSD requires the integrity of mPFC pyramidal neurons to elicit prosocial effects.

The behavioral outcomes of LSD on sociability are mediated by AMPA and 5-HT_2A_ receptors. The role of these two receptors has been described in studies showing that 5-HT_2A_ receptor stimulation by LSD produces EPSPs in layer V of the mPFC, thus promoting a release of glutamate in the apical dendritic region of layer V pyramidal cells in the PFC. These effects are reversed by the specific antagonism of the AMPA receptor (11). In addition, LSD (100 nM) increases the excitatory response of interneurons in the piriform cortex following AMPA (5 µM) application in patch-clamp experiments (10).

We showed that mTORC1 in excitatory neurons is necessary for the LSD-induced prosocial effects. The lack of prosocial effects caused by LSD in *Raptor*^*f/f*^*/Camk2α-Cre* mice was also paralleled by a reduced 5-HT_2A_/AMPA excitatory response following the microiontophoretic application of DOI and quisqualate to mPFC neurons in mutant mice treated with LSD. Intriguingly, a dysregulation of mTORC1 is associated with neurodevelopmental disorders such as ASD (3), characterized by an impairment in SB and in the activation of mTORC1.

Similarly, ketamine, a nonpsychedelic hallucinogenic drug, elicits antidepressant effects through mTORC1. The acute administration of a subanesthetic dose of ketamine induces strong antidepressant effects in rats via the activation of mTOR signaling in the PFC. Indeed,, these effects are prevented by the intracranial administration of rapamycin (24). This indicates that the mTOR pathway could exert a pivotal role in orchestrating the behavioral effects of hallucinogenic drugs. Unlike ketamine, we found that LSD does not have antidepressant properties in naïve mice, in contrast to recent work showing that LSD at 150 μg/kg produced antidepressant-like effects 5 wk after a single administration (35). This discrepancy might be due to the different species (rat vs. mouse) employed, and to the fact that LSD might require a longer time frame to induce antidepressant-specific molecular changes and neuronal plasticity. Further studies are needed to decipher the multifaceted behavioral properties of LSD, the optimal dosage, and its long-term effects.

It is noteworthy that the behavioral outcomes documented in our study were obtained with a relatively low dose of LSD, compared to previous studies in animals (100 to 200 µg/kg) (36, 37) and humans (38). We have previously demonstrated that relatively low doses (5 to 20 µg/kg) of LSD decrease the activity of serotonergic neurons originating in the dorsal raphe nucleus (DRN) with no effects on dopaminergic neurons in the ventral tegmental area (VTA), while higher doses (60 to 120 µg/kg) inhibit dopaminergic neurons in the VTA (39). Such high doses have been correlated with psychotic-like hallucinogenic symptoms such as hyperlocomotion, stereotypical behavior, head twitches, and impaired prepulse inhibition in rodents (36, 37), thus leading us and others to hypothesize that low doses might exert behavioral benefits without psychotic-like adverse reactions. For instance, 3,4-methylenedioxy-methamphetamine (MDMA), a psychoactive compound chemically similar to psychedelics, has prosocial effects at low doses via serotonergic neurotransmission which is distinct from the stimulation of dopaminergic signaling observed only at high doses, which mediates the reward pathway (15).

In conclusion, our study unveils a mechanism contributing to the prosocial effects of LSD, through the stimulation of 5-HT_2A_ and AMPA receptors and the activation of the mTORC1 pathway in excitatory neurons. A limitation of this study is that our results do not mirror the outcomes observed in a clinical setting where an acute LSD dose (100 to 200 μg) is effective in increasing empathy (4). The lack of effect on sociability after acute LSD administration in rodents might be due to the fact that empathy cannot be easily measured in animal models of social interaction. Moreover, while LSD and psychedelics induce rapid tolerance (40–42), we did not observe tolerance after 7 d. The lack of tolerance is conceivably due to the very low doses of LSD used over a short time period. Despite these limitations, our study should pave the path for future preclinical and clinical investigations, which could improve pharmacotherapies for pathologies linked to SB dysfunction for which there are no resolutive pharmacological treatments.

## Materials and Methods

A full description of all experimental procedures is provided in *SI Appendix*.

### Animals and Housing.

Inbred C57BL/6J male mice, mutant *Raptor*^*f/f*^*:Camk2α-Cre* and *Raptor*^*f/f*^*:Gad-Cre* male mice and their control littermates (8 to 12 wk old, weighing 25 to 30 g) were used. Male Sprague–Dawley rats (weighing 250 to 300 g) were purchased (Charles River) and used for the DSI test in rats. For DSI and TCT in mice, intruder male C57BL/6J mice (weighing 25 to 30 g) were used. Animals were housed in standard polycarbonate cages under standard laboratory conditions (12-h light–dark cycle, lights on at 07:30 and off at 19:30; temperature 20 ± 2 °C; 50 to 60% relative humidity). To target excitatory neurons, floxed mice (*Raptor*^*f/f*^) on a C57BL/6J background were crossed with *Camk2α-Cre* mice to generate *Raptor*^*f/f*^*:Camk2α-Cre* mice. To target inhibitory neurons, floxed mice (*Raptor*^*f/f*^) were crossed with *Gad-Cre* to generate *Raptor*^*f/f*^*:Gad-Cre* mice. For details concerning the generation of floxed mice (*Raptor*^*f/f*^), please refer to ref. 43. All experimental procedures were conducted in accordance with the guidelines set by the Canadian Institutes of Health Research for Animal Care and Scientific Use and the McGill University Animal Care Committee (protocol no. 5764). All experiments were conducted during the light phase, between 9:00 and 15:00.

### Drugs.

LSD, 8-OH-DPAT, MDL, NBQX, DOI, NMDA, and quisqualic acid were used. For details concerning suppliers, solubility, and regimen of administration, please see *SI Appendix*.

### Viral Constructs.

Adeno-associated viruses (AAV-CamKIIα-Arch.3.0-eYFP) or a control vector expressing eYFP (AAV-CamKIIα-eYFP) were obtained from the Neurophotonics Centre, Québec (QC), Canada with the permission of Karl Deisseroth’s laboratory, Stanford University, Stanford, CA.

### In Vivo Electrophysiology.

In vivo electrophysiological recordings in both IL and PL regions of the mPFC were performed according to our standardized protocols. For details, please see *SI Appendix*.

### Behavioral Procedures.

Separate cohorts of mice received single or repeated LSD and intra-mPFC microinfusion of Veh, NBQX, or MDL. Thirty minutes after the acute injection, mice were subjected to the DSI. The same cohort of mice underwent the DSI 24 h later. The separated cohort of mice treated with repeated LSD administration or Veh were subjected to the DSI 24 h after the last injection, and then underwent the TCT and OFT. Another cohort of mice underwent NSFT while a final cohort underwent the FST and SPT. Another cohort of mice treated with Veh or LSD underwent the DSI coupled with optogenetic inhibition. *Raptor*^*f/f*^*:Camkk2α-Cre*, *Raptor*^*f/f*^*:Gad-Cre* and their control littermates treated with Veh or repeated LSD, underwent the DSI and TCT. A cohort of rats treated with repeated LSD administration underwent the DSI. Details concerning the behavioral procedures are reported in *SI Appendix*.

### Optogenetic Experiments.

Optogenetic manipulations were combined with in vivo electrophysiology or behavioral testing procedures. For details about the optical fiber implantation, optogenetic manipulation, and histological verification of viral expression, please see *SI Appendix*.

### Western Blot.

Male mice treated with repeated LSD (*n* = 6/group) were killed and brains were dissected for Western blot analysis in the PFC. Experimental procedures, antibody dilutions, and uncropped Western blot images are provided in *SI Appendix* and in *SI Appendix*, Table S1.

### Immunohistochemistry.

For details about immunohistochemistry in control, *Raptor*^*f/f*^*:Camk2α-Cre*, and *Raptor*^*f/f*^*:Gad-Cre* adult male mice, please see *SI Appendix*.

### Statistical Analyses.

Data are expressed as mean ± SEM and were analyzed using GraphPad Prism 9 (GraphPad Software Inc.). Statistical results are detailed in *SI Appendix*, Table S2. When comparing two datasets, unpaired Student’s *t* tests were performed; when variance was significantly different between groups, Welch’s correction was used. Regular and repeated measures one- and two-way ANOVA were used considering these factors: treatment and genotype for behavioral tests, treatment and current for microiontophoresis, virus and light on/off for optogenetic-electrophysiology experiments, and virus and treatment, or treatment and light on/off, for optogenetic-behavioral experiments. Three-way ANOVA was used employing treatment, genotype, and current as factors for microiontophoresis experiments in mutant mice. When appropriate, post hoc tests with Bonferroni multiple comparison corrections were also performed to examine differences among groups. Values of *P* < 0.05 were considered significant.

## Supplementary Material

Supplementary File

## Data Availability

All study data are included in the article and supporting information.
