# Supplementary material for: Lysergic acid diethylamide (LSD) promotes social behavior through mTORC1 in the excitatory neurotransmission
Source: Proc Natl Acad Sci U S A. 2021 Jan 25;118(5):e2020705118. doi: 10.1073/pnas.2020705118 (PMC7865169; doi:10.1073/pnas.2020705118)
Supplement: Supplementary File [file pnas.2020705118.sapp.pdf]

PNAS
www.pnas.org

Supplementary Information for

Lysergic acid diethylamide (LSD) promotes social behavior through mTORC1 in the excitatory neurotransmission

Danilo De Gregorio, Jelena Popic, Justine P. Enns, Antonio Inserra, Agnieszka Skalecka, Athanasios Markopoulos, Luca Posa, Martha Lopez-Canul, He Qianzi, Christopher K. Lafferty, Jonathan P. Britt, Stefano Comai, Argel Aguilar-Valles, Nahum Sonenberg and Gabriella Gobbi

*Corresponding authors emails: danilo.degregorio@mcgill.ca, nahum.sonenberg@mcgill.ca and gabriella.gobbi@mcgill.ca

This PDF file includes:

Supplementary materials and methods

Figures S1 to S9

Tables S1 to S2

SI References

SUPPLEMENTARY MATERIALS AND METHODS

Drugs

Lysergic diethylamide (LSD) (Sigma-Aldrich, London, UK), 8-hydroxy-2-(di-n-propylamino) tetralin hydrobromide (8-OH-DPAT), (Sigma-Aldrich, Oakville, Canada) were dissolved in a 0.9% NaCl vehicle (veh) solution and administered intraperitoneally (i.p.) at a volume of 10 ml/kg body weight. For intra-mPFC infusions, (R)-(+)-α-(2,3-Dimethoxyphenyl)-1-[2-(4-fluorophenyl) ethyl]-4-piperinemethanol (MDL 100 907) (Tocris Bioscience, Missouri, USA) and 2,3-dihydroxy-6-nitro-7-sulfamoyl-benzo[f]quinoxaline-2,3-dione (NBQX) were initially dissolved in water containing 1.5–5 μl of glacial acetic acid to make a 10 mM stock solution. They were then further diluted in artificial cerebrospinal fluid (aCSF, 125 mM NaCl, 2.5 mM KCl, 1.26 mM CaCl2, 1.18 mM MgCl2) and administered in a final volume of 1 μL. For microiontophoretic applications, the following drugs were used: the 5-HT_{1A} receptor agonist 8-hydroxy-N,N-dipropyl-2-aminotetralin HBr (8-OH-DPAT, 50 mM in 200 mM NaCl, pH 4.0–4.5), the 5-HT_{2A} receptor agonist (±)-2,5-Dimethoxy-4-iodoamphetamine (DOI, 10 mM in 200 mM NaCl, pH 4.0–4.5), N-methyl-D-aspartate (NMDA, 50 mM in 200 mM NaCl, pH 8) and the AMPA agonist quisqualic acid (1.5 mM in 400 mM NaCl, pH 8.0). Urethane (Sigma-Aldrich, Oakville, Canada) was used as an anesthetic in all non-recovery electrophysiological experiments.

Single and repeated LSD treatment

For single administration experiments, mice were injected with vehicle (veh) or LSD (30 μg/kg) and were tested 30 min and 24 h after the injection. This relatively low dose for an animal study was chosen because it decreases the 5-HT firing activity of the Dorsal Raphe Nucleus (DRN) without affecting the dopaminergic neurons of the Ventral Tegmental Area (VTA) (1). This low dose does not produce stereotypies, does not affect locomotion, nor does it stimulate the dopaminergic system as LSD does when administered at high doses (2). For repeated administration, mice and rats received LSD (30 μg/kg/day, i.p., for 7 days) and were tested 24 h after the last injections for behavioral, electrophysiological and biomolecular evaluation.

***In Vivo* Electrophysiology**

Preparation for recording procedures.

In vivo single-unit extracellular recordings were performed following our standardized protocols (3-5). Firstly, the mice were anesthetized with urethane (1.4 g/kg, intraperitoneal), then mounted on a stereotaxic frame (David Kopf Instruments, Tujunga, California) with the skull positioned horizontally. Anesthesia was confirmed by the absence of nociceptive reflex reaction to a tail or paw pinch and lack of eye blink response to pressure. Body temperature was maintained at 37 ± 0.5 C throughout the experiment using a thermistor-controlled heating pad (Seabrook Medical Instrument, Inc., Seabrook, NH, USA). Recordings were carried out using microiontophoresis multi-barreled (Harvard/Applied Scientific Instrumentation, OR, USA) glass micropipettes pulled on a Narashige (Tokyo, Japan) PE-2 pipette puller. The micropipettes were preloaded with fiberglass strands to promote capillary filling with 2% Pontamine Sky Blue solution in 2M NaCl, and their tips were broken down to diameters of 1–3 mm for single-barreled and 10–15 mm for multi-barreled recordings. The impedances ranged from 2 to 6MΩ. The stereotaxic brain coordinate system by Paxinos and Franklin (6) was used in all electrophysiological experiments. Using a hydraulic micropositioner (model 650; David Kopf Instruments, Tujunga, California), the electrode was advanced slowly into the brain structure at approximately 0.15 mm/min to minimize the probability of missing slow-spiking neurons. To maximize sampling without introducing considerable tissue damage, three to five electrode descents were performed. Single-unit activity was recorded as discriminated action potentials amplified by a Tennelec (Oakridge, TN) TB3 MDA3 amplifier, post-amplified and filtered by a Realistic 10 band frequency equalizer, digitized by a CED1401 interface system (Cambridge Electronic Design, Cambridge, UK), processed online, and analyzed off-line using Spike2 software version 5.20 for Windows PC (Microsoft, Seattle, WA). A Npi electronic Gmbh Microiontophoretic System (Tamm, Germany) was used for local (iontophoretic) drug applications. The spontaneous single-spike activity of neurons was recorded for at least 2 min; the first 30 s immediately after detecting the neuron was not considered to eliminate mechanical artifacts due to electrode displacement. For experiments requiring acute drug injection, a catheter was inserted intraperitoneally prior to electrophysiological recording to facilitate intraperitoneal administration. Drug response was considered inhibitory if the drug decreased the basal firing rate of a neuron by at least 10%. At the end of each recording session, the recording site was marked by iontophoretic ejection (1–10 mA,

negative current for 10min) of Pontamine Sky Blue for later histological verification of recording sites. All recordings were carried out between 1400 and 2200 hours. A first cohort of mice (n=4) underwent *in vivo* electrophysiology combined with optogenetic photo-inhibition in the mPFC. Another group of mice treated with a single dose of LSD (30 μg/kg, i.p.) underwent single unit extracellular recordings of pyramidal neurons in the mPFC. Then mice treated with repeated LSD (30 μg/kg/day, i.p., for 7 days) underwent single unit extracellular recordings of pyramidal neurons in the mPFC as reported in Supplementary. *Raptor$^{f/f}$:Camk2α-Cre* and their littermates treated with LSD (30 μg/kg/day, i.p., for 7 days) or veh underwent *in vivo* electrophysiological recordings in the mPFC. A total of 33 mice were used for these experiments

Extracellular recordings and microiontophoresis from the medial prefrontal cortex

This procedure was performed according to our protocols (7, 8). The multi-barreled micropipette was lowered into the prelimbic (PL) and infralimbic (IL) region of the ventromedial prefrontal cortex (mPFC) (1.5–2.0 mm anterior to bregma; 2.5–3.5 mm ventral to the dura mater; 0.25 mm from the midline, within layer 5 of the cortex). The side barrels had impedances ranging from 50 to 150 MΩ and contained NMDA, 8-OH-DPAT, DOI, or quisqualic acid, and a NaCl solution (2 M) for automatic current balancing. As most of the pyramidal neurons are not spontaneously active under anesthesia, prolonged low-current NMDA ejections (-5nA) were introduced to activate them within their physiological firing rates (0.5–10 Hz in the mPFC) (7, 9, 10). It has been shown that there is no response difference between pharmacologically induced, and spontaneously firing pyramidal neurons (9). Neurons were also identified based on their steady response to standard short pulses of NMDA and by large amplitude, long duration, and single-action potential patterns alternating with complex spike discharges (7, 8, 11). Employing increasing currents (-20 to -50 nA, 30 s currents), NMDA was also used to assess the sensitivity of this receptor. DOI was used to assess the sensitivity of 5-HT_{2A} receptors and was ejected as an anion (-20 to -50 nA, 30 s currents) and retained with a current of 20 nA. Quisqualate was used to assess the sensitivity of α-amino-3-hydroxy-5-methyl-4-isoxazole propionate (AMPA) receptor and was ejected as an anion (-20 to -50 nA, 30 s currents) and retained with a current of 20 nA. 8-OH-DPAT was used to assess the sensitivity of 5-HT_{1A} receptors and was ejected as a cation (+20, +30 and +50 nA in the mPFC, 30 s currents) and retained with a current of -20nA. Pyramidal activity was identified by large amplitudes (0.5–1.2 mV), long durations (0.8–1.2 ms), and

as single action potentials alternating with complex spike discharges. Pyramidal neural response to systemic or microiontophoretic drug application was expressed as percentage increase/decrease from pre-drug microionthophoretic application baseline (0 nA current). For bursting analysis, cells exhibiting 3 consecutive spikes with inter-spike intervals < 45 ms were classified as burst-firing cells (12).

Behavioral Tests

Direct Social Interaction (mice)

This test was performed according to our standardized protocol (13). Test mice were placed in a clean cage and given 10 min to habituate. Immediately after habituation, a novel age- and sex-matched conspecific stranger mouse was introduced into the cage and the mice were able to freely engage in social interaction for 10 min. The interaction time, defined by the following behaviors: nose-to-anogenital sniffing, nose-to-nose sniffing, and social grooming, was manually scored.

Direct Social Interaction (rats)

This test was adapted from our protocol (14). After receiving repeated administration with veh or LSD (30 μg/kg/day, for 7 days), Sprague Dawley rats were placed in an open field arena (80 x 80 x1 5 cm) and given 10 min to habituate. Immediately after habituation, a novel age- and sex-matched conspecific stranger rat was introduced into the cage and the rats were able to freely engage in social interaction for 10 min. The interaction time, defined by the following behaviors: nose-to-anogenital sniffing, nose-to-nose sniffing, and social grooming, was manually scored. The test was performed 24 hours later the last injection of LSD or veh.

Three-Chamber Test

This test was performed according to our standardized protocol (13, 15). A three-chamber arena with openings between the chambers was used to assess sociability and preference for social novelty. Test mice were placed in the middle chamber and allowed to freely explore the empty three-chamber arena for 10 min. Immediately after habituation, an unfamiliar mouse (stranger 1, male C57BL/6J, age matched) was introduced into 1 of the 2 side chambers, enclosed in a wire cage, thus allowing only the test mouse to

initiate social interaction. An identical empty wire cage was placed in the other side chamber. With this setup, the test mouse was again placed in the middle chamber and allowed to explore the three-chamber arena for 10 min. At the end of the 10-min sociability test, a new unfamiliar mouse (stranger 2, male C57BL/6J, age matched) was placed in the previously unoccupied wire cage. The test mouse was observed for an additional 10 min to assess social novelty (as explained below). The location of the empty wire cage was alternated between side chambers for different test mice to prevent chamber biases. Stranger 1 (S1) and 2 (S2) mice were always taken from separate home cages and counterbalanced for each side of the chamber apparatus and stranger cage. The time spent interacting with S1, S2, or the empty cage, was manually scored. The interaction time was determined by measuring the duration of the head/body contacts or climbing of the subject mouse upon either the empty cage or the cage containing the stranger mouse In order to ensure a comparisons across treatments, strangers and genotype groups, and also to reduce the impact of variable exploration times between mice, we used a “sociability index” for each mouse, calculated as: 100 × (S1 interaction time− empty cage interaction time)/(S1 interaction time + empty cage interaction time) and a “social novelty index” for each mouse, calculated as: 100 × (S2 interaction time− S1 interaction time)/(S2 interaction time + S1 interaction time)(16). Number of contacts upon the empty cage or the cage containing the S1/S2 mouse were also included, as well as the percentage of preference for the empty cage (empty cage interaction time/ (empty cage interaction time+ S1 interaction time) x 100) during the sociability phase. Mice were excluded from further analysis if they either failed to explore the empty cage or the mouse S1/S2 chambers, or if they spent more than 75% of the allotted time in the center chamber, not exploring either chambers containing S1, S2, or the empty cage. For these reasons, 19 mice out of 190 undergoing the TCT were excluded (171 in total). All stranger mice were purchased from Charles River Laboratories (Sherbrooke, QC)

Open field activity testing

According to our protocols (7), mice were individually placed at the center of a white-painted open field arena (40 x 40 x 15 cm) and left to explore the whole arena for 20 min. The experiment took place under standard room lighting (350 lx); a white lamp (100 W) was suspended 2 m above the arena. Anxiety-like

thigmotactic (‘wall-following’) behavior was measured by the frequency and total duration of central zone (30 x 30 cm) visits. Other ethological measures analyzed included grooming, rearing and locomotor activity.

Novelty-suppressed feeding test

According to our protocols (4), this procedure was used to measure novelty-induced anxiety-like behavior (neo-hypophagia, the inhibition of feeding upon exposure to an anxiogenic novel environment). This test has been widely used to validate the acute effects of putative anxiolytics. The mice were food-deprived for 48 h, then each mouse was placed in a brightly illuminated (100 W, 350 lx) open arena (40 x 40 cm, white-painted floor with walls 30 cm high, containing standard lab chow (3 pellets) on the floor at the center of the arena. The latency to initiate feeding (in seconds) was noted and used as an index of anxiety -like behavior. The cut-off time was 600s (Feeding latency was also observed in the home cage containing 3 pellets spread on the floor to exclude the possibilities that mice were not hungry; the session was terminated immediately after the mice initiated feeding).

Forced swim test

According to our protocol (17), mice were individually placed into Plexiglas cylindrical bins (20 cm diameter, 50 cm high) filled with water (25–27 °C) to a depth of 20 cm. This depth did not allow the tail or the hind paws to touch the bottom of the bin. Mice were allowed to swim for 6 min. Infrared light-sensitive CCD cameras allowed for the capture and storage of images with the videotrack system (View Point Life Science, Montreal (QC), Canada)). After recording, mice were rescued using a plastic grid and placed in a cage near a heat lamp to dry. The behavioral tracking system was calibrated so that a mouse was considered immobile when making only minimal movements necessary to keep its head above the water. The total duration of activity was determined during the last 4 min.

Sucrose Preference Test

This test was performed employing our protocols (17). Mice were individually housed 3 days before the beginning of the test. They were then trained for 3 days to consume water from two bottles. During these 3 days, the two bottles containing water were replaced for 1 h a day with two bottles filled with a 2% (w/v)

sucrose solution. Next, mice were subjected to a 48h procedure during which they were allowed to discriminate and select between 2 drinking bottles, one containing water and the other the sucrose solution. To avoid conditioned place preference learning, the bottles were placed on the home cage for 48 h (starting at the beginning of the light phase, 7:00 A.M.) and their positions were interchanged in the mid-point of each light (1:00 P.M) and dark (1:00 A.M.) cycle over these 2 days. The sucrose preference (%) was determined as follows: sucrose solution intake (g)/total fluid intake (g) × 100.

Viral vector infusion and optical fiber implantation

For the optogenetic procedure, mice were anesthetized with isoflurane and their skulls secured on a stereotaxic frame. A single craniotomy was performed and a Hamilton syringe (Reno, NV) fitted with a 28-gauge needle was used to place a viral bolus (1 µl) at the following coordinates in millimeters within the mPFC (AP: 1.9, ML: 0.4, DV −2.5), and were infused with AAV-CamKIIα-Arch.3.0-EYFP or a control vector expressing eYFP (AAV-CamKIIα-eYFP). 22 days later, mice were prepared for electrophysiological recordings and a unilateral optic fiber cannula (0.7 mm center-to-center, 200 µM, 0.22 NA, Doric, Quebec) was lowered above the viral bolus using the following coordinates (AP:1.9, ML:1, DV: -2.47, with an angle of 20°). For the behavioral experiments, the optical fiber was implanted 15 days post viral injection. Fibers were fixed to the skull using dental cement and a pair of skull screws.

Cannula implantation for intra-mPFC infusion

The intra-mPFC antagonist infusions experiments followed the same procedure as above. Mice were implanted with internal cannulae (Plastics One, HRS Scientific, Canada) above the mPFC extending 2 mm below the cannulae guides as explained in Supplemental. Microinfusions of aCSF, NBQX or MDL occurred at a rate of 0.1 ml/min over 2 minutes, once per day, for 7 days, 10 min before the systemic injection of LSD or veh (saline). To maximize diffusion, the internal cannulae were kept in place for an additional 2 minutes after the infusion. 24 hours after the last infusion, locomotor impairment was assessed in the OFT. Immediately after the OFT, mice underwent the DSI and the TCT. The experimental groups were divided

as following: (i) intracortical MDL or NBQX and i.p. LSD, (ii) intracortical aCSF and i.p. LSD, (iii) intracortical MDL 100 907 or NBQX and i.p. veh (saline); (iv) intracortical aCSF and i.p. veh (saline).

Optogenetic manipulations

Photo-inhibition was performed employing a modified protocol (18). For *in vivo* electrophysiology recordings of mPFC pyramidal neurons, C57BL/6J mice were anesthetized and placed on a stereotaxic frame. Fiberoptic patchcords (200 μM, 0.22 NA, Doric, Quebec) and mono fiberoptic cannulae were connected to a dual laser diode (LD) fiber light source (450nm-75 mW/520nm-60 mW, Doric Lenses, Canada) and lowered into the mPFC together with a multi-barreled recording electrode as mentioned before. After isolating a neuron, mice transfected with AAV-CamKIIα-Arch.3.0-eYFP or AAV-CamKIIα-eYFP received green light (530 nm laser, intensity 10–12 mW at the optical fiber tip) for 10 s. The Arch3.0 opsin is an improved variant of Arch, resulting in larger light activated currents compared to Arch. For more details, please refer to (19). For behavioral experiments, another cohort of mice treated with LSD or veh for 7 days were tested for DSI. In this case, each animal coupled to a fiberoptic patch cord was connected to a fiberoptic rotary joint (Doric, Quebec). The rotary joint was connected via the patch cord to the dual LD fiber light source for optogenetic inhibition. Mice underwent habituation to the patch cord in their home cage 5 min per day, for two days. The last habituation was performed 24 hours before the experiment. The day of the test, both the control group and LSD-treated animals (last LSD dose 24 hours before the test) were connected to the laser and, after 10 min of habituation, they underwent the DSI and received the green light (intensity, 10 mW; 530-nm laser) for 10 min (continuous light). The light was delivered immediately after the introduction of the intruder mouse and it lasted for the duration of the test. The same cohort of mice underwent the DSI again 24 hours later (using different intruder mice coming from different cages) with the light turned OFF. This inhibition protocol has been demonstrated not to bleach or injure brain tissue (20, 21).

Histological verification of viral expression

At the end of each optogenetic experiment, animals were anesthetized with a ketamine/xylazine (120 mg/kg/10 mg/kg) cocktail and transcardially perfused with 4% paraformaldehyde in phosphate-buffered saline. Brains were removed and fixed in 4% paraformaldehyde for 6–12 h; they were then placed in 30% sucrose for 48–72 h before freezing. For viral expression verification, brains were sectioned into 25-µm slices (Leica VT1000s) and mounted with MOWIOL plus DAPI solution (Sigma-Aldrich, Oakville, Ontario, Canada). mPFC sections (PL and IL) were imaged on a confocal microscope (LSM710, Zeiss, McGill University Cell Imaging and Analysis Network).

Western Blotting.

Western blot were performed following our standardized protocols (15). Frontal cortex tissue (from male mice, 8-12 weeks old, weighing 25–30 g) was homogenized in RIPA buffer (50 mM Tris-HCl pH 7.4, 150 mM NaCl, 1% NP-40, 0.1% SDS, 0.5% sodium deoxycholate, 5 mM EDTA pH 8.0, 1 mM EGTA pH 8.0, 10 mM NaF, 1 mM β-glycerophosphate and 1 mM sodium orthovanadate) containing protease inhibitors (Roche). Protein extracts were denatured with heat and 15 µg of protein extracts were resolved by SDS–PAGE Following electrophoresis, proteins were transferred to nitrocellulose membranes and western blotting was performed. Membranes were stripped in 25 mM glycine-HCl pH 2.0 and 1% SDS for 3 x 10 min at room temperature, followed by 3 x 10 min washing in TBST before reprobing. Immunoreactivity was detected by enhanced chemiluminescence (plus-ECL, PerkinElmer, Inc.) after exposure to X-ray film (Denville Scientific, Inc.). Quantification of immunoblots was performed using ImageQuant 5.2. Values were normalized against glyceraldehyde 3-phosphate dehydrogenase (GAPDH) levels.

Full-length immunoblots are shown in Supplementary Figures 7-9. For details concerning the used antibodies and dilutions see Supplementary Table S1.

Immunohistochemistry

Control, *Raptorf/f:Camk2α-Cre* and *Raptorf/f:Gad-Cre* adult male mice (8-12 weeks old) underwent transcardial fixation using phosphate-buffered saline (PBS) and then 4% paraformaldehyde. Brains were

cryopreserved, flash frozen and stored at -80 °C until processing. Coronal slices (25 μm) were prepared and blocked for 1 h (room temperature) in 5% Goat serum (GS) and 0.5% Triton-X in PBS. Then, slices were incubated overnight (4 °C) in 1% GS in PBS with the antibodies: rabbit anti-phospho RPS6 (Ser 240/244), mouse anti-CaMKII-α, mouse anti-Gad67. Next, sections were incubated in darkness (1 h, room temperature) in blocking buffer with AlexaFluor 488 goat anti-rabbit IgG (Thermo Fisher Scientific, 1:400), AlexaFluor 546 goat anti-mouse IgG (Thermo Fisher Scientific, 1:400), and DAPI (4',6-Diamidino-2-Phenylindole, Dihydrochloride, Life Technologies, 1:1000). Sections were washed with PBS (3 times, 5 min each) after each incubation step. Samples were visualized using the Zeiss LSM800 laser scanning confocal microscope. For details, please see the supplemental section.

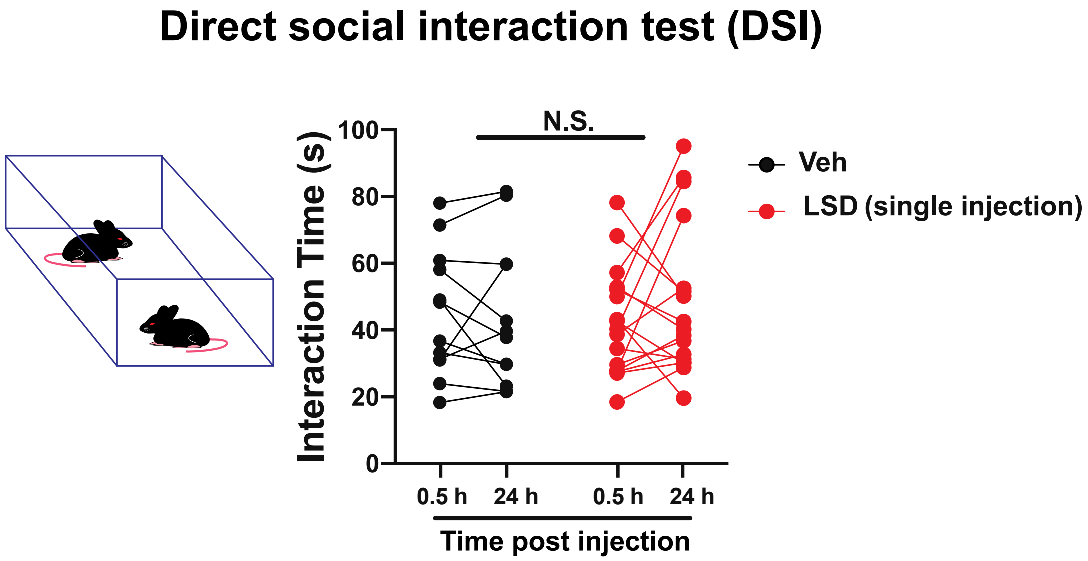

Fig. S1. Single administration of LSD does not affect direct social interaction in mice.

Mice treated acutely with veh or LSD (30 μg/kg) and tested 30 min later in the direct social interaction test displayed the same interaction time toward a stranger conspecific. The same cohort of mice underwent the same test using a different intruder mouse 24 later. N=12-15 mice per group. Two-way ANOVA for repeated measures. N.s.=not significant. For statistical details, please see supplementary table 2.

Direct social interaction test (DSI) in rats

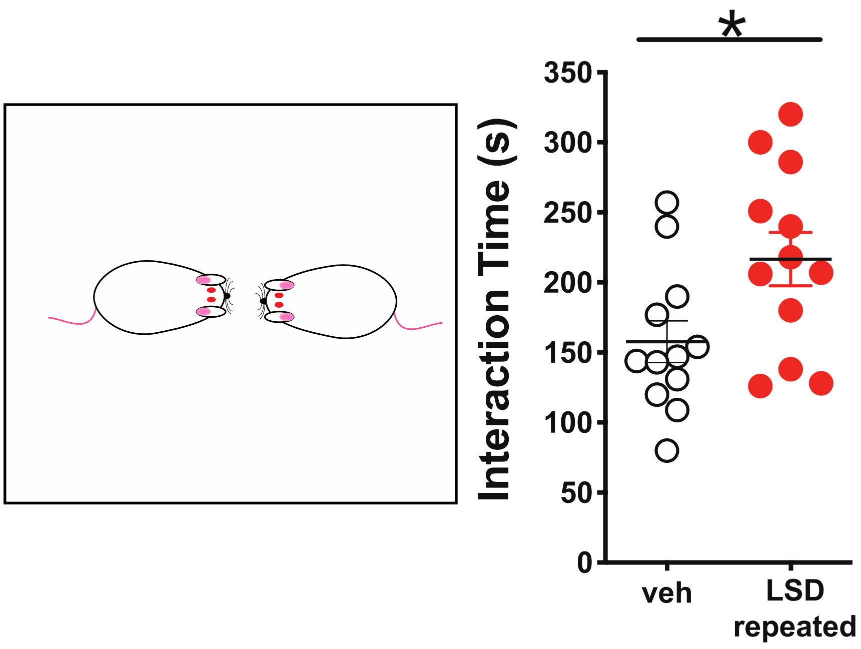

Fig. S2. Repeated LSD administration increases direct social interaction in Sprague-Dawley rats. Repeated LSD increased the time spent interacting in adult rats, compared to veh. N=12 rats per group. Each line represents mean ± SEM and each point represents the time spent interacting for each rat. Student unpaired 2-tailed t test. *P<0.05. For statistical details, please see supplementary table 2.

Novelty suppressed feeding test (NSFT)

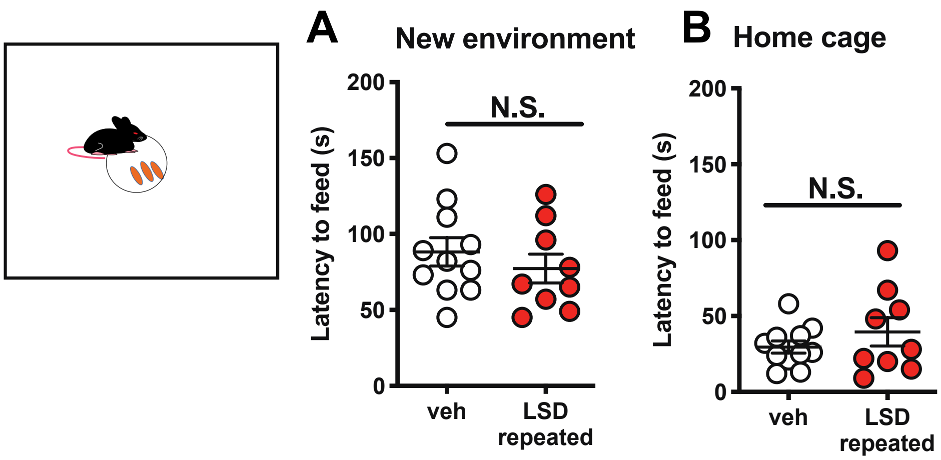

Fig. S3. Repeated LSD does not affect stereotypic and anxiety-like behavior in mice.

(A) LSD did not affect the latency to feed in the novel environment or in the (B) home cage in the novelty suppressed feeding test. N=9-10 mice per group). Each line represents mean ± SEM and each point represents a single mouse value. Student unpaired 2-tailed t test. N.S.=not significant. For statistical details, please see supplementary table 2.

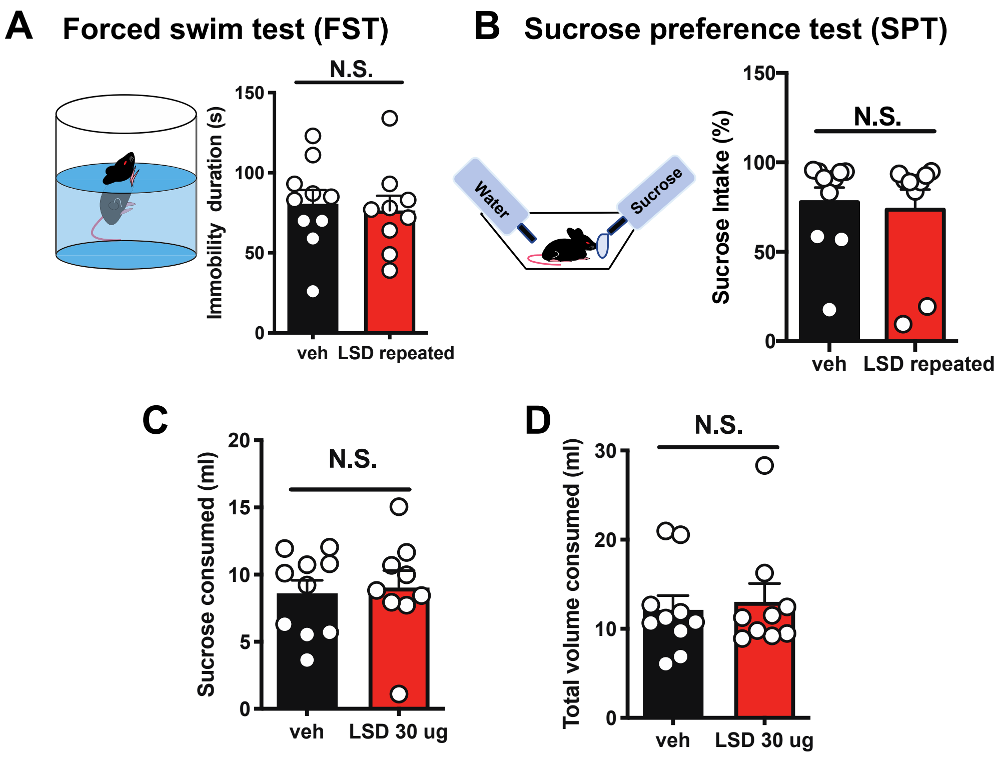

Fig. S4. Repeated LSD does not affect depressive-like behavior in mice.

(A) Repeated LSD administration did not induce any changes (A) in the immobility duration in the forced swim test. Moreover, in the sucrose preference test (SPT, no statistical difference was detected in the sucrose intake percentage (B), neither in the amount of sucrose (C) or total volume consumed (D). N=9/10 mice per group. Each bar represents mean ± SEM and each point represents the individual result of each mouse. Student unpaired 2-tailed t test. N.S.= not significant. For statistical details, please see supplementary table 2.

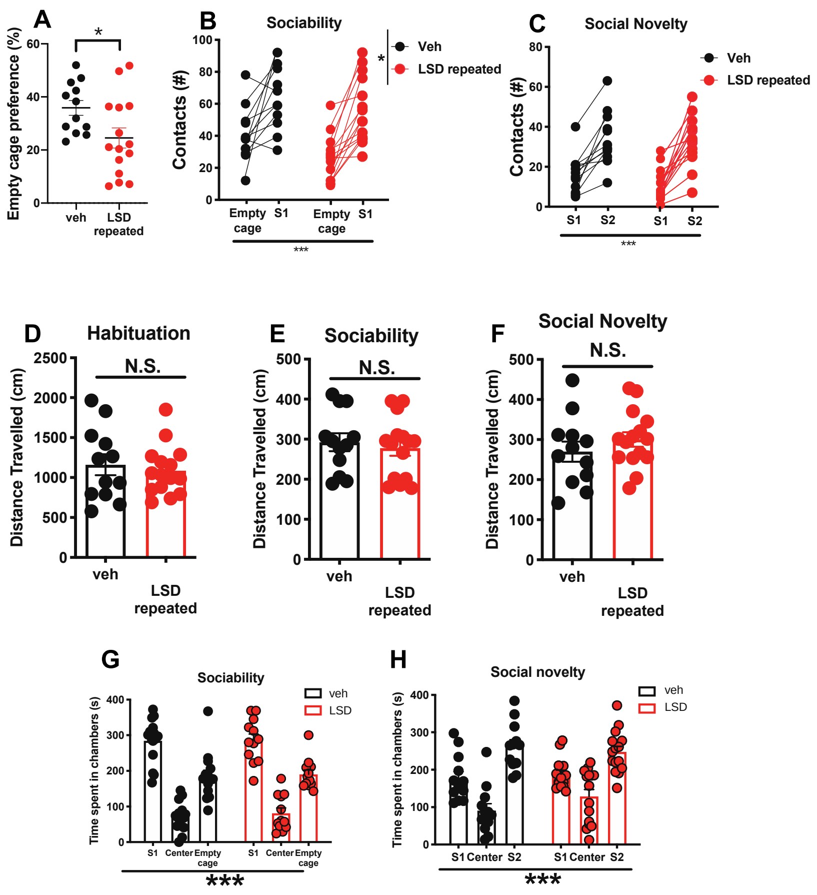

Fig. S5. Further behavioral assessments during the TCT.

(A) Mice treated with LSD showed less percentage preference for the empty cage and increased number of contacts with the wire cage containing the S1 (B) or the S2 (C) mouse during the sociability or the social novelty phase, respectively. Moreover, no statistical difference was detected in the distance travelled when mice were allowed to explore the three-chamber apparatus in the 10 minutes of habituation (D), in the sociability (E) and in the social novelty (F) phase. Repeated LSD administration did not affect the time spent in each chamber during the sociability (G) or the social novelty (H) phase of the TCT. *p<0.05, **p<0.01, ***p<0.001. Unpaired t-test, One-way ANOVA followed by Bonferroni post-hoc comparisons, two-way ANOVA. N.S.= not significant. For statistical details, please see supplementary table 2.

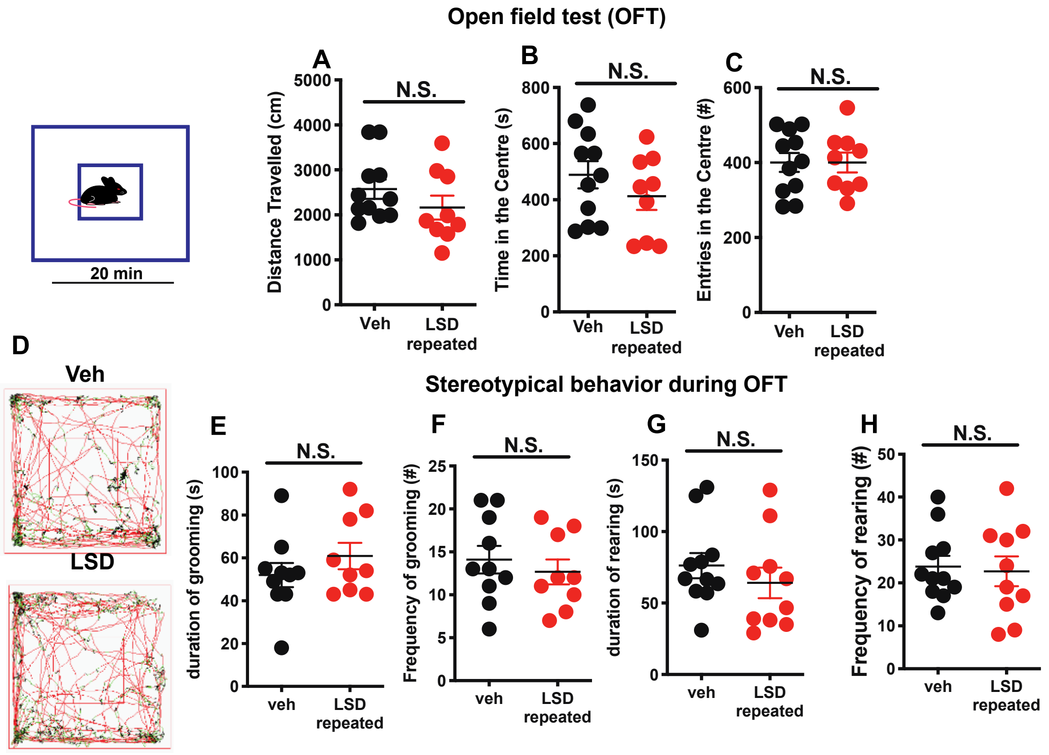

Fig. S6. Repeated LSD does not affect stereotypic and anxious-like behavior in mice.

(A) LSD did not affect the distance travelled, (B) time spent in the center and (C) the frequency of visits to the center area of the open field test (OFT). (D) Example of horizontal movement traces in the OFT of mice treated with veh (left) or LSD (right). Repeated LSD administration did not induce any changes in the duration of (E) and in the frequency (F) of grooming, neither in the duration (G) and in the frequency (H) of rearing during the open field test..N=9-10 mice per group). Student unpaired 2-tailed t test. N.S.= not significant. For statistical details, please see supplementary table 2.

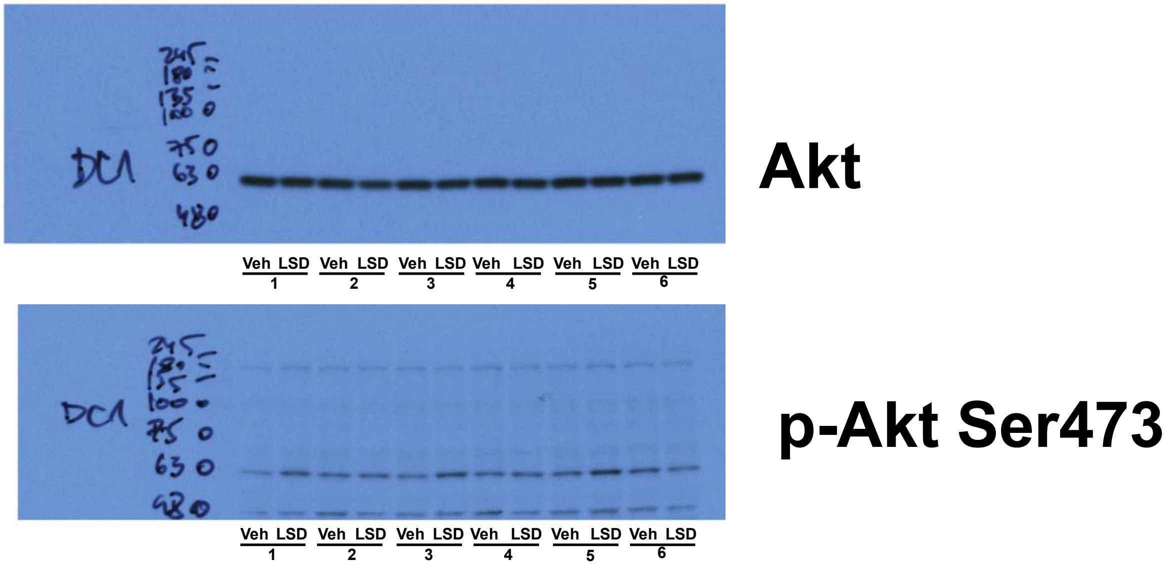

Fig. S7. Uncropped blot images of total Akt (top) and phosphorylated (bottom) Akt Ser473 levels.

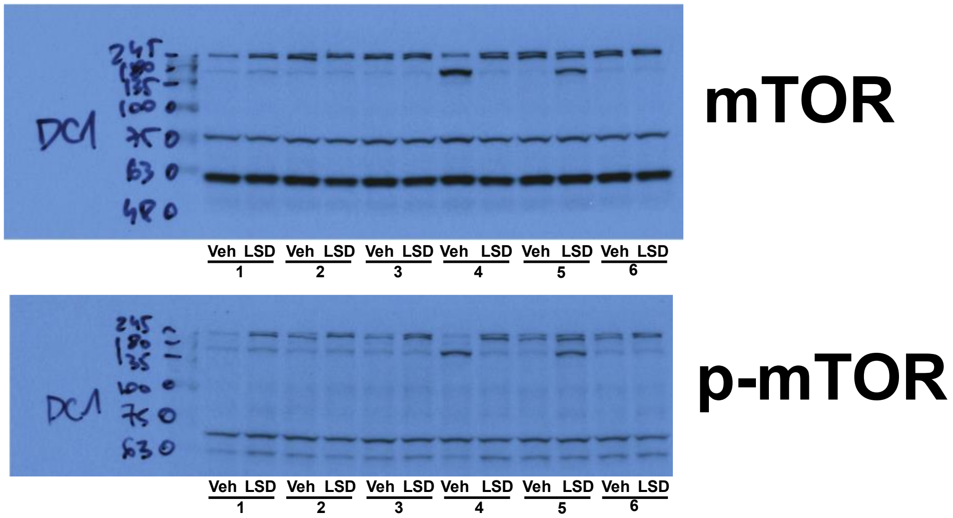

Fig. S8. Uncropped blot images of total (top) and phosphorylated (bottom) mTOR levels

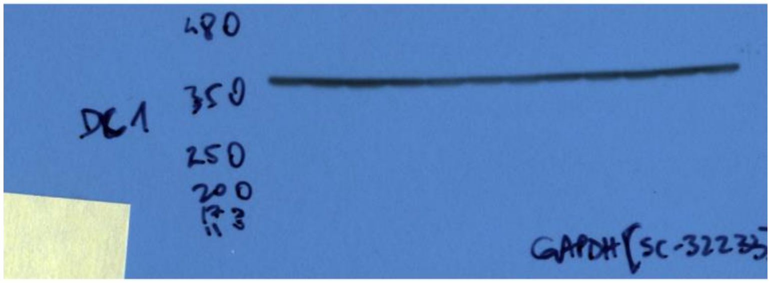

GAPDH

Fig. S9. Uncropped blot images of GAPDH

Antibody	Catalog number/manufacturer	Dilution
Rabbit anti Akt	4691, Cell Signaling	1:1000
Rabbit anti Phospho-Akt (Ser473)	9271, Cell Signaling	1:1000
Rabbit anti mTOR	2972, Cell Signaling	1:1000
Rabbit anti Phospho-mTOR	2971, Cell Signaling	1:1000
Anti-rabbit IgG	NA 934, GE Healthcare	1:10000
Anti-mouse IgG	NA 931, GE Healthcare	1:10000
Mouse anti GAPDH	sc-32233, Santa Cruz	1:10000
Rabbit phospho-RPS6 (Ser 240/244)	5364, Cell Signaling	1:800
Mouse anti-CaMKII-α	50049, Cell Signaling	1:1000
Mouse anti-Gad67	MAB5406, EMD Millipore	1:1000
Alexa Fluor 488 goat anti-rabbit IgG	A11034, Thermo Fisher Scientific	1:400
Alexa Fluor 546 goat anti-mouse IgG	A10036, Thermo Fisher Scientific	1:400
DAPI (4',6-Diamidino-2-Phenylindole, Dihydrochloride)	D1306, Life Technologies	N/A

Table S1. Antibody information related to experimental procedures, with catalog number/manufacturer, and used dilutions. N/A= not applicable.

<table>
<tr><th>Figure</th><th>Panel</th><th>Test</th><th>Group-size</th><th>Statistic</th><th>P value</th><th>Pair-wise comparison</th><th>Statistic 2</th></tr>
<tr><td rowspan="5">1</td><td>B</td><td>Student unpaired 2-tailed t test</td><td>Veh: 12 mice; LSD=15 mice</td><td>t=2.200, df=25</td><td>P=0.0372</td><td>N/A</td><td>N/A</td></tr>
<tr><td>D</td><td>Repeated measures 2-way ANOVA (cage x treatment)</td><td>Veh: 12 mice; LSD= 15 mice</td><td>Interaction:F (1, 25) = 4.281

Treatment:F (1, 25) = 0.8134

Cage:F (1, 25) = 58.88</td><td>P=0.0490

P=0.3651

P<0.0001</td><td>Bonferroni post hoc comparison</td><td><table>
<tr><th>Test details</th><th>t</th><th>P value</th></tr>
<tr><td>Empty cage vs S1: veh</td><td>3.76</td><td><0.01</td></tr>
<tr><td>Empty cage vs S1: LSD</td><td>7.307</td><td><0.001</td></tr>
<tr><td>S1:veh vs S1:LSD</td><td>0.6912</td><td>ns</td></tr>
</table></td></tr>
<tr><td>E</td><td>Student unpaired 2-tailed t test</td><td>Veh: 12 mice; LSD=15 mice</td><td>t=2.299, df=25</td><td>P=0.0302</td><td>N/A</td><td>N/A</td></tr>
<tr><td>G</td><td>Repeated measures 2-way ANOVA (cage x treatment)</td><td>Veh: 12 mice; LSD= 15 mice</td><td>Interaction:F (1, 25) = 8.170

Treatment:F (1, 25) = 2.706

Cage:F (1, 25) = 74.73</td><td>P=0.0085

P=0.1125

P<0.0001</td><td>Bonferroni post hoc comparison</td><td><table>
<tr><th>Test details</th><th>t</th><th>P value</th></tr>
<tr><td>S1 vs S2: veh</td><td>3.444</td><td><0.01</td></tr>
<tr><td>S1 vs S2: LSD</td><td>7.654</td><td><0.001</td></tr>
<tr><td>S2:veh vs S2:LSD</td><td>3.074</td><td><0.05</td></tr>
</table></td></tr>
<tr><td>H</td><td></td><td>Veh: 12 mice; LSD=15 mice</td><td>t=2.139, df=2</td><td>P=0.0424</td><td>N/A</td><td>N/A</td></tr>
</table>

		Student unpaired 2-tailed t test					
	K	Student unpaired 2-tailed t test	5 recordings from 5 mice	t=0.008302, df=8	0.9936	N/A	N/A
	M	Student unpaired 2-tailed t test	Veh:12 recordings from 5 mice; LSD: 16 recordings from 5 mice	t=0.2490, df=26	0.8053	N/A	N/A
	O	Student unpaired 2-tailed t test	Veh:12 recordings from 5 mice; LSD: 16 recordings from 5 mice	t=2.587, df=18	0.0186	N/A	N/A
	P	Repeated measures 2-way ANOVA (treatment x current)	Veh:12 recordings from 5 mice; LSD: 16 recordings from 5 mice	Interaction: F (3, 78) = 0.3012<br>Current: F (3, 78) = 4.648<br>Treatment : F (1, 26) = 0.07862	P=0.8244<br>P=0.0048<br>P=0.7814	N/A	N/A
	Q	Repeated measures 2-way ANOVA (treatment x current)	Veh:12 recordings from 5 mice; LSD: 16 recordings from 5 mice	Interaction:F (3, 75) = 2.861	P=0.0424	Bonferroni post hoc comparison	**Test details** / **t** / **P value**<br>Veh 0 vs. Veh -20 / 2.719 / <0.05<br>Veh 0 vs. Veh -30 / 4.701 / <0.001<br>Veh 0 vs. Veh -50 / 5.221 / <0.001<br>Veh-20 vs. Veh-30 / 1.982 / ns

				Treatment: F (3, 75) = 46.59	P<0.0001		Veh- 20 vs. Veh-50	2.502	ns
							Veh- 30 vs.Veh -50	0.5199	ns
							LSD 0 vs. LSD -20	4.709	<0.001
							LSD 0 vs. LSD -30	9.253	<0.001
							LSD 0 vs. LSD -50	10.28	<0.001
				Current: F (1, 25) = 14.31	P=0.0009		LSD-20 vs. LSD-30	4.544	<0.001
							LSD- 20 vs. LSD-50	5.576	<0.001
							LSD- 30 vs.LSD -50	1.031	ns
							Veh 0 vs LSD 0	0	ns
							Veh -20 vs LSD -2-	1.266	ns
							Veh -30 vs LSD -30	3.175	<0.01
							Veh -50 vs LSD - 5-	3.533	<0.01
	**R**	Repeated measures 2-way ANOVA (treatment x current)	Veh:12 recordings from 5 mice; LSD: 16 recordings from 5 mice	Interaction: F (3, 75) = 3.035	P=0.0343	Bonferroni post hoc comparison	**Test details**	**t**	**P value**
							Veh 0 vs. Veh-20	2.133	ns
							Veh 0 vs. Veh-30	2.566	ns
							Veh 0 vs. Veh-50	3.89	<0.01
							Veh-20 vs. Veh-30	0.4332	ns
							Veh-20 vs. Veh-50	1.758	ns
				Treatment: F (2.159, 53.97) = 27.05	P<0.0001		Veh-30 vs. Veh-50	1.325	ns
							LSD 0 vs. LSD-20	6.203	<0.001
							LSD 0 vs. LSD-30	6.487	<0.001
							LSD 0 vs. LSD -50	9.008	<0.001
							LSD -20 vs. LSD-30	0.2832	ns
							LSD-20 vs. LSD-50	2.804	<0.05
				Current:F (1, 25) = 29.43	P<0.0001		LSD-30 vs. LSD-50	2.521	ns
							Veh 0 vs LSD 0	N/A	ns
							Veh -20 vs LSD-20	2.704	ns

							Veh -30 vs LSD-30	4.143	<0.01
							Veh-50 vs LSD-50	3.899	<0.01
	**S**	Repeated measures 2-way ANOVA (treatment x current)	Veh:12 recordings in 5 mice;<br>LSD: 16 recordings in 5 mice	Interaction: F (3, 18) = 0.4704<br>Current: F (3, 18) = 5.883<br>Treatment: F (1, 6) = 1.565	P=0.7066<br>P=0.0055<br>P=0.2576	N/A	N/A		
	U	2-way ANOVA (intra-mPFC x treatment	Veh:19 mice;<br>LSD: 20 mice;<br>MDL+LSD:13;<br>NBQX+LSD:13	Interaction: F (2, 87) = 0.0328	P=0.0328	Bonferroni post hoc comparison	**Test details**	**t**	**P value**
							Veh:ACSF vs. Veh:MDL	0.4174	ns
							Veh:ACSF vs. Veh:NBQX	0.4093	ns
							Veh:ACSF vs. LSD :ACSF	3.5	<0.05
							Veh:ACSF vs. LSD :MDL	0.2536	ns
							Veh:ACSF vs. LSD :NBQX	0.5395	ns
				treatment: F (1, 87) = 0.078	P=0.0780		Veh:MDL vs. Veh:NBQX	0.00757	ns
							Veh:MDL vs. LSD :ACSF	3.64	<0.01
							Veh:MDL vs. LSD :MDL	0.1448	ns
							Veh:MDL vs. LSD :NBQX	0.1224	ns
				intra-mPFC: F (2, 87) = 3.556	P=0.0050		Veh:NBQX vs. LSD :ACSF	3.631	<0.01
							Veh:NBQX vs. LSD :MDL	0.1374	ns
							Veh:NBQX vs. LSD :NBQX	0.1298	ns
							LSD :ACSF vs. LSD :MDL	3.403	<0.05
							LSD :ACSF vs. LSD :NBQX	3.692	<0.01
							LSD :MDL vs. LSD :NBQX	0.2623	ns
	V	2-way ANOVA (intra-mPFC x treatment	Veh:19 mice;<br>LSD: 20 mice;	Interaction: F (2, 61) = 0.0551	P=0.0551	Bonferroni post hoc comparison	**Test details**	**t**	**P value**
							Veh:ACSF vs. Veh:MDL	0.5951	ns

			MDL+LSD:13; NBQX+LSD:13				Veh:ACSF vs. Veh:NBQX	0.03261	ns
							Veh:ACSF vs. LSD :ACSF	3.2	<0.05
							Veh:ACSF vs. LSD :MDL	0.08078	ns
							Veh:ACSF vs. LSD :NBQX	0.2147	ns
				treatment: F (1, 61) = 3.817	P=0.0553		Veh:MDL vs. Veh:NBQX	0.5632	ns
							Veh:MDL vs. LSD :ACSF	3.724	<0.01
							Veh:MDL vs. LSD :MDL	0.5035	ns
							Veh:MDL vs. LSD :NBQX	0.342	ns
				intra-mPFC: F (2, 61) = 4.381	P=0.0167		Veh:NBQX vs. LSD :ACSF	3.232	<0.05
							Veh:NBQX vs. LSD :MDL	0.04889	ns
							Veh:NBQX vs. LSD :NBQX	0.1845	ns
							LSD :ACSF vs. LSD :MDL	3.21	<0.05
							LSD :ACSF vs. LSD :NBQX	3.177	<0.05
							LSD :MDL vs. LSD :NBQX	0.1356	ns
	W	2-way ANOVA (intra-mPFC x treatment	Veh:19 mice; LSD: 20 mice; MDL+LSD:13; NBQX+LSD:13	Interaction: F (2, 61) = 5.086	P=0.0091	Bonferroni post hoc comparison	**Test details**	**t**	**P value**
							Veh:ACSF vs. Veh:MDL	0.2372	ns
							Veh:ACSF vs. Veh:NBQX	0.3612	ns
							Veh:ACSF vs. LSD :ACSF	3.55	<0.05
							Veh:ACSF vs. LSD :MDL	0.05252	ns
							Veh:ACSF vs. LSD :NBQX	0.1987	ns
				treatment: F (1, 61) = 2.202	P=0.1430		Veh:MDL vs. Veh:NBQX	0.1161	ns
							Veh:MDL vs. LSD :ACSF	3.235	<0.05
							Veh:MDL vs. LSD :MDL	0.2837	ns
							Veh:MDL vs. LSD :NBQX	0.4152	ns
				intra-mPFC: F (2, 61) = 3.657	P=0.0316		Veh:NBQX vs. LSD :ACSF	3.189	<0.05
							Veh:NBQX vs. LSD :MDL	0.4058	ns

							Statistic 2		
							Veh:NBQX vs. LSD :NBQX	0.5331	ns
							LSD :ACSF vs. LSD :MDL	3.525	<0.05
							LSD :ACSF vs. LSD :NBQX	3.486	<0.05
							LSD :MDL vs. LSD :NBQX	0.1461	ns

Figure	Panel	Test	Group-size	Statistic	P value	Pair-wise comparison	Statistic 2		
							**Test details**	**t**	**P value**
2	C	Repeated measures 2-way ANOVA (virus x light ON-OFF)	eYFP= 7 recordings from 2 mice; Arch3.0= 5 recordings from 2 mice	Interaction:F (1, 10) = 29.30	P=0.0003	Bonferroni post hoc comparison	Light On - Light OFF		
				Virus (eYFP vs Arch3.0)t: F (1, 10) = 1.680	P=0.2240		eYFP	0.104	ns
				Light (On vs Off) : F (1, 10) = 27.87	P=0.0004		Arch 3.0	6.999	<0.0001
							**Test details**	**t**	**P value**
	F	2-way ANOVA (treatment x virus)	eYFP+veh= 13 mice; eYFP+LSD= 13 mice; Arch3.0+ veh= 14 mice; Arch3.0+LSD= 13 mice	Interaction:F (1, 49) = 4.346	P=0.0423	Bonferroni post hoc comparison	Veh:Eyfp vs. Veh:Arch 3.0	3.089	<0.05
				Virus (eYFP vs Arch3.0): F (1, 49) = 3.658	P=0.0616		Veh:Eyfp vs. LSD 30:Eyfp	2.801	<0.05
							Veh:Eyfp vs. LSD 30:Arch 3.0	3.153	<0.05
				Treatment : F (1, 49) = 41.12	P<0.0001		Veh:Arch 3.0 vs. LSD 30:Eyfp	5.941	<0.001
							Veh:Arch 3.0 vs. LSD 30:Arch 3.0	0.1227	ns

						LSD 30:Eyfp vs. LSD 30:Arch 3.0	5.955	<0.001
	G	Repeated measure 2-way ANOVA (treatment x light ON-OFF)	Arch3.0+ veh= 14 mice; Arch3.0+LSD= 13 mice	Interaction:F (1, 25) = 15.96<br>Treatment : F (1, 25) = 3.139<br>Light : F (1, 25) = 77.47	P=0.0005<br>P=0.0886<br>P<0.0001	Bonferroni post hoc comparison	**Test details**	**t**	**P value**
							Light ON - Light OFF<br>Veh<br>LSD repeated	 3.464<br>8.885	 <0.02<br><0.001
							Veh - LSD repeated<br>Light ON<br>Light OFF	 0.4034<br>3.496	 ns<br><0.01
**Figure**	**Panel**	**Test**	**Group-size**	**Statistic**	**P value**	**Pair-wise comparison**	**Statistic 2**		
3	A	Student unpaired 2-tailed t test	Veh:6 mice; LSD:6 mice	t=1.089, df=10	0.3017	N/A	N/A		
	B	Student unparied 2-tailed t test	Veh:6 mice; LSD:6 mice	t=2.408, df=10	0.0368	N/A	N/A		
	C	Student unpaired 2-tailed t test	Veh:6 mice; LSD:6 mice	t=0.6422, df=10	0.5352	N/A	N/A		

	D	Student unpaired 2-tailed t test	Veh:6 mice; LSD:6 mice	t=7.825, df=10	P<0.0001	N/A	N/A
	G	Student unpaired 2-tailed t test with Welch's correction	Raptor +/+:Gad2-Cre= 44 cells in 3 mice<br><br>Raptor f/f:Gad2-Cre= 33 cells in 3 mice	t=6.071, df=44.06	P<0.0001	N/A	NA
	H	2-way ANOVA (treatment x genotype)	Raptor +/+:Gad2-Cre+veh=8 mice; Raptor +/+:Gad2-Cre+LSD=8 mice; Raptor f/f:Gad2-Cre+veh=8 mice; Raptor f/f:Gad2-Cre+LSD=8 mice	Interaction:F (1, 28) = 0.01752<br><br>Treatment: F (1, 28) = 22.62<br><br>Genotype: F (1,28) = 0.2420	P=0.8956<br><br>P<0.0001<br><br>P=0.6266	NA	NA
	I	2-way ANOVA (treatment x genotype)	Raptor +/+:Gad2-Cre+veh=8 mice; Raptor +/+:Gad2-Cre+LSD=8 mice; Raptor f/f:Gad2-Cre+veh=8 mice;	Interaction:F (1, 28) = 0.01198<br><br>Treatment: F (1, 28) = 6.698	P=0.7318<br><br>P=0.0151	NA	NA

			Raptor f/f:Gad2-Cre+LSD=8 mice	Genotype: F (1,28) = 0.9674	P=0.3338		
	J	2-way ANOVA (treatment x genotype)	Raptor +/+:Gad2-Cre+veh=8 mice; Raptor +/+:Gad2-Cre+LSD=8 mice; Raptor f/f:Gad2-Cre+veh=8 mice; Raptor f/f:Gad2-Cre+LSD=8 mice	Interaction:F (1, 28) = 0.4143<br>Treatment: F (1, 28) = 16.01<br>Genotype: F (1,28) = 4.066	P=0.5251<br>P=0.0004<br>P=0.0534	NA	NA

**Figure**	**Panel**	**Test**	**Group-size**	**Statistic**	**P value**	**Pair-wise comparison**	**Statistic 2**
4	B	Student unpaired 2-tailed t test with Welch's correction	Raptor +/+:Camk2α= 35 cells in 3 mice<br>Raptor f/f:Camk2α= 36 cells in 3 mice	t=7.508, df=34.06	P<0.0001	N/A	NA
	c	2-way ANOVA (treatment x genotype)	Raptor +/+:Camk2α-Cre+veh=12 mice; Raptor +/+:Camk2α-	Interaction:F (1, 43) = 4.228	P=0.0459	Bonferroni post hoc comparison	**Test details** / t / P value<br>veh:Raptor +/+:Camk2α-Cre vs. veh:Raptor f/f:Camk2α-Cre / 0.6639 / ns

			Cre+LSD=12 mice; Raptor f/f:Camk2α-Cre+veh=13 mice; Raptor f/f:Camk2α-Cre+LSD=10 mice	Treatment: F (1, 43) = 10.87	P=0.0020		veh:Raptor +/+:Camk2α-Cre vs. LSD:Raptor +/+:Camk2α-Cre	3.844	<0.01
							veh:Raptor +/+:Camk2α-Cre vs. LSD:Raptor f/f:Camk2α-Cre	0.2291	ns
				Genotype: F (1, 43) = 8.779	P=0.0049		veh:Raptor f/f:Camk2α-Cre vs. LSD:Raptor +/+:Camk2α-Cre	4.584	<0.001
							veh:Raptor f/f:Camk2α-Cre vs. LSD:Raptor f/f:Camk2α-Cre	0.8651	ns
							LSD:Raptor +/+:Camk2α-Cre vs. LSD:Raptor f/f:Camk2α-Cre	3.436	<0.01
	D	2-way ANOVA (treatment x genotype)	Raptor +/+:Camk2α-Cre+veh=12 mice; Raptor +/+:Camk2α-Cre+LSD=12 mice; Raptor f/f:Camk2α-Cre+veh=13 mice; Raptor f/f:Camk2α-Cre+LSD=10 mice	Interaction:F (1, 43) = 5.580	P=0.0228	Bonferroni post hoc comparison	**Test details**	**t**	**P value**
							veh:Raptor +/+:Camk2α-Cre vs. veh:Raptor f/f:Camk2α-Cre	0.4259	ns
				Treatment: F (1, 43) = 4.127	P=0.0484		veh:Raptor +/+:Camk2α-Cre vs. LSD:Raptor +/+:Camk2α-Cre	3.154	<0.05
							veh:Raptor +/+:Camk2α-Cre vs. LSD:Raptor f/f:Camk2α-Cre	0.6245	ns
				Genotype: F (1, 43) = 8.667	P=0.0052		veh:Raptor f/f:Camk2α-Cre vs. LSD:Raptor +/+:Camk2α-Cre	3.643	<0.01
							veh:Raptor f/f:Camk2α-Cre vs. LSD:Raptor f/f:Camk2α-Cre	0.2303	ns
							LSD:Raptor +/+:Camk2α-Cre vs. LSD:Raptor f/f:Camk2α-Cre	3.632	<0.01

								Test details	t	P value
	E	2-way ANOVA (treatment x genotype)	Raptor +/+:Camk2α-Cre+veh=12 mice; Raptor +/+:Camk2α-Cre+LSD=12 mice; Raptor f/f:Camk2α-Cre+veh=13 mice; Raptor f/f:Camk2α-Cre+LSD=10 mice	Interaction:F (1, 43) = 8.154	P=0.0066	Bonferroni post hoc comparison	veh:Raptor +/+:Camk2α-Cre vs. veh:Raptor f/f:Camk2α-Cre		0.09625	ns
				Treatment: F (1, 43) = 2.916	P=0.0949		veh:Raptor +/+:Camk2α-Cre vs. LSD:Raptor +/+:Camk2α-Cre		3.276	<0.05
							veh:Raptor +/+:Camk2α-Cre vs. LSD:Raptor f/f:Camk2α-Cre		0.8758	ns
				Genotype: F (1, 43) = 8.922	P=0.0046		veh:Raptor f/f:Camk2α-Cre vs. LSD:Raptor +/+:Camk2α-Cre		3.437	<0.01
							veh:Raptor f/f:Camk2α-Cre vs. LSD:Raptor f/f:Camk2α-Cre		0.7999	ns
							LSD:Raptor +/+:Camk2α-Cre vs. LSD:Raptor f/f:Camk2α-Cre		3.999	<0.01
	G	2-way ANOVA (treatment x genotype)	Raptor +/+:Camk2α-Cre+veh=15 recordings from 4 mice; Raptor +/+:Camk2α-Cre+LSD=14 recordings from 4 mice;	Interaction:F (1, 58) = 0.5223	P=0.4728	N/A	N/A			
				Treatment: F (1, 58) = 0.1283	P=0.7215					
				Genotype: F (1, 58) = 1.251	P=0.2679					

			Raptor f/f:Camk2α-Cre+veh=16 recordigs from 5 mice;<br>Raptor f/f:Camk2α-Cre+LSD=17 recordings from 5 mice				
	H	Repeated measures 3-way ANOVA (current x treatmentx genotype)	Raptor +/+:Camk2α-Cre+veh=15 recordings from 4 mice;<br>Raptor +/+:Camk2α-Cre+LSD=14 recordings from 4 mice;<br>Raptor f/f:Camk2α-Cre+veh=16 recordigs from 5 mice;<br>Raptor f/f:Camk2α-Cre+LSD=17 recordings from 5 mice	current:F (1.999, 114.0) = 37.48<br><br>genotype:F (1, 57) = 24.98<br><br>treatment: F (1, 57) = 58.81<br><br>current x genotype: F (3, 171) = 6.151<br><br>current x treatment: F (3, 171) = 16.02<br><br>genotype x treatment:	P<0.0001<br><br>P<0.0001<br><br>P<0.0001<br><br>P=0.0005<br><br>P<0.0001<br><br>P=0.0023	N/A	N/A

				F (1, 57) = 10.19<br><br>current x genotype x treatment: F (3, 171) = 1.987	P=0.1178		
	I	Repeated measures 3-way ANOVA (current x treatment x genotype)	GROUP A) Raptor +/+:Camk2α-Cre+veh=15 recordings from 4 mice; (GROUP B) Raptor +/+:Camk2α-Cre+LSD=14 recordings from 4 mice; (GROUP C) Raptor f/f:Camk2α-Cre+veh=16 recordigs from 5 mice; (GROUP D) Raptor f/f:Camk2α-Cre+LSD=17 recordings from 5 mice			Bonferroni post hoc comparison	See Test details table below

Test details	t	P value
0:Group A vs. 0:Group B	0	ns
0:Group A vs. 0:Group C	3.11E-15	ns
0:Group A vs. 0:Group D	0	ns
0:Group A vs. -20:Group A	0.5131	ns
0:Group A vs. -20:Group B	8.656	<0.0001
0:Group A vs. -20:Group C	0.4621	ns
0:Group A vs. -20:Group D	2.077	ns
0:Group A vs. -30:Group A	3.044	ns
0:Group A vs. -30:Group B	11.6	<0.0001
0:Group A vs. -30:Group C	1.594	ns
0:Group A vs. -30:Group D	6.376	<0.0001
0:Group A vs. -50:Group A	4.353	0.0103
0:Group A vs. -50:Group B	11.61	<0.0001
0:Group A vs. -50:Group C	2.367	ns
0:Group A vs. -50:Group D	7.439	<0.0001
0:Group B vs. 0:Group C	4.73E-15	ns

							0:Group B vs. 0:Group D	3.1E-15	ns
							0:Group B vs. -20:Group A	0.5252	ns
							0:Group B vs. -20:Group B	8.716	<0.0001
							0:Group B vs. -20:Group C	0.4688	ns
							0:Group B vs. -20:Group D	2.106	ns
							0:Group B vs. -30:Group A	3.116	ns
							0:Group B vs. -30:Group B	11.69	<0.0001
							0:Group B vs. -30:Group C	1.617	ns
							0:Group B vs. -30:Group D	6.465	<0.0001
							0:Group B vs. -50:Group A	4.456	0.0084
							0:Group B vs. -50:Group B	11.69	<0.0001
							0:Group B vs. -50:Group C	2.402	ns
							0:Group B vs. -50:Group D	7.544	<0.0001
							0:Group C vs. 0:Group D	0	ns
							0:Group C vs. -20:Group A	0.509	ns
							0:Group C vs. -20:Group B	8.511	<0.0001
							0:Group C vs. -20:Group C	0.451	ns
							0:Group C vs. -20:Group D	2.046	ns
							0:Group C vs. -30:Group A	3.02	ns
							0:Group C vs. -30:Group B	11.41	<0.0001
							0:Group C vs. -30:Group C	1.556	ns
							0:Group C vs. -30:Group D	6.279	<0.0001
							0:Group C vs. -50:Group A	4.318	0.0128
							0:Group C vs. -50:Group B	11.41	<0.0001
							0:Group C vs. -50:Group C	2.311	ns
							0:Group C vs. -50:Group D	7.326	<0.0001
							0:Group D vs. -20:Group A	0.4999	ns
							0:Group D vs. -20:Group B	8.354	<0.0001

							0:Group D vs. -20:Group C	0.4469	ns
							0:Group D vs. -20:Group D	1.994	ns
							0:Group D vs. -30:Group A	2.966	ns
							0:Group D vs. -30:Group B	11.2	<0.0001
							0:Group D vs. -30:Group C	1.541	ns
							0:Group D vs. -30:Group D	6.122	<0.0001
							0:Group D vs. -50:Group A	4.241	0.0161
				Current:F (3, 174) = 65.58	P<0.0001		0:Group D vs. -50:Group B	11.2	<0.0001
							0:Group D vs. -50:Group C	2.29	ns
							0:Group D vs. -50:Group D	7.143	<0.0001
				Genotype:F (1, 58) = 43.09	P<0.0001		-20:Group A vs. -20:Group B	8.131	<0.0001
							-20:Group A vs. -20:Group C	0.04694	ns
							-20:Group A vs. -20:Group D	1.577	ns
				Treatment: F (1, 58) = 158.8	P<0.0001		-20:Group A vs. -30:Group A	2.531	ns
							-20:Group A vs. -30:Group B	11.08	<0.0001
							-20:Group A vs. -30:Group C	1.085	ns
				Current x genotype:	P=0.0045		-20:Group A vs. -30:Group D	5.876	<0.0001
				F (3, 171) = 4.513			-20:Group A vs. -50:Group A	3.84	0.0481
							-20:Group A vs. -50:Group B	11.08	<0.0001
							-20:Group A vs. -50:Group C	1.858	ns
				Current x treatment:	P<0.0001		-20:Group A vs. -50:Group D	6.939	<0.0001
				F (3, 171) = 17.59			-20:Group B vs. -20:Group C	8.042	<0.0001
							-20:Group B vs. -20:Group D	6.248	<0.0001
							-20:Group B vs. -30:Group A	5.54	0.0002

				Genotype x treatment:	P=0.0001		-20:Group B vs. -30:Group B	2.97	0.5631
				F (1, 58) = 16.69			-20:Group B vs. -30:Group C	6.894	<0.0001
							-20:Group B vs. -30:Group D	1.889	ns
							-20:Group B vs. -50:Group A	4.2	0.0163
				Current x genotype x treatment:	P=0.0183		-20:Group B vs. -50:Group B	2.973	ns
				F (3, 174) = 3.430			-20:Group B vs. -50:Group C	6.109	<0.0001
							-20:Group B vs. -50:Group D	0.8104	ns
							-20:Group C vs. -20:Group D	1.599	ns
							-20:Group C vs. -30:Group A	2.558	ns
							-20:Group C vs. -30:Group B	10.94	<0.0001
							-20:Group C vs. -30:Group C	1.105	ns
							-20:Group C vs. -30:Group D	5.832	0.0001
							-20:Group C vs. -50:Group A	3.856	0.0515
							-20:Group C vs. -50:Group B	10.94	<0.0001
							-20:Group C vs. -50:Group C	1.86	ns
							-20:Group C vs. -50:Group D	6.879	<0.0001
							-20:Group D vs. -30:Group A	0.8883	ns
							-20:Group D vs. -30:Group B	9.094	<0.0001
							-20:Group D vs. -30:Group C	0.5042	ns
							-20:Group D vs. -30:Group D	4.127	0.022
							-20:Group D vs. -50:Group A	2.164	ns
							-20:Group D vs. -50:Group B	9.097	<0.0001
							-20:Group D vs. -50:Group C	0.2439	ns
							-20:Group D vs. -50:Group D	5.148	0.0008
							-30:Group A vs. -30:Group B	8.489	<0.0001
							-30:Group A vs. -30:Group C	1.426	ns
							-30:Group A vs. -30:Group D	3.41	ns

-30:Group A vs. -50:Group A	1.309	ns
-30:Group A vs. -50:Group B	8.492	<0.0001
-30:Group A vs. -50:Group C	0.6524	ns
-30:Group A vs. -50:Group D	4.474	0.0073
-30:Group B vs. -30:Group C	9.794	<0.0001
-30:Group B vs. -30:Group D	4.735	0.0013
-30:Group B vs. -50:Group A	7.149	<0.0001
-30:Group B vs. -50:Group B	0.00302	ns
-30:Group B vs. -50:Group C	9.009	<0.0001
-30:Group B vs. -50:Group D	3.657	0.0826
-30:Group C vs. -30:Group D	4.738	0.0022
-30:Group C vs. -50:Group A	2.725	ns
-30:Group C vs. -50:Group B	9.797	<0.0001
-30:Group C vs. -50:Group C	0.755	ns
-30:Group C vs. -50:Group D	5.785	0.0001
-30:Group D vs. -50:Group A	2.135	ns
-30:Group D vs. -50:Group B	4.738	0.0027
-30:Group D vs. -50:Group C	3.989	0.0359
-30:Group D vs. -50:Group D	1.021	ns
-50:Group A vs. -50:Group B	7.152	<0.0001
-50:Group A vs. -50:Group C	1.951	ns
-50:Group A vs. -50:Group D	3.198	ns
-50:Group B vs. -50:Group C	9.012	<0.0001
-50:Group B vs. -50:Group D	3.66	0.0498
-50:Group C vs. -50:Group D	5.037	0.0007

Figure	Panel	Test	Group-size	Statistic	P value	Pair-wise comparison	Statistic 2

**S 1**	**N/A**	2-way ANOVA (treatment x time)	Veh:12 mice; LSD= 17 mice;	Interaction:F(1,27)=. 0.9739<br><br>Treatment:F (1, 27) = 0.01606<br><br>Time:F(1,27)=0.4893	P=0.3325<br><br>P=0.9001<br><br>P=0.4902	N/A	NA
**S 2**	N/A	Student unpaired 2-tailed t test	Veh: 12 rats; LSD=12 rats	t=2.441, df=22	0.0231	N/A	NA
**S3**	A	Student unpaired 2-tailed t test	Veh: 10 mice; LSD=9 mice	t=0.8260, df=18	0.4196	N/A	NA
	B	Student unpaired 2-tailed t test	Veh: 10 mice; LSD=9 mice	t=1.044, df=18	0.101	N/A	NA
**S4**	A	Student unpaired 2-tailed t test	Veh: 10 mice; LSD=9 mice	t=0.07076, df=17	0.9444	N/A	NA
	B		Veh: 10 mice; LSD=9 mice	t=0.3112, df=17	0.7594	N/A	NA

		Student unpaired 2-tailed t test					
	C	Student unpaired 2-tailed t test	Veh: 10 mice; LSD=9 mice	t=0.2830, df=17	0.7806	N/A	NA
	D	Student unpaired 2-tailed t test	Veh: 10 mice; LSD=9 mice	t=0.3433, df=17	0.7356	N/A	NA
**S5**	A	Student unpaired 2-tailed t test	Veh: 12 mice; LSD=15 mice	t=2.299, df=25	0.0302	N/A	N/A
	B	Repeated measures 2-way ANOVA (cage x treatment)	Veh: 12 mice; LSD=15 mice	Interaction:F (1, 25) = 0.4109<br>Treatment:F (1, 25) = 5.194<br>Cage:F (1, 25) = 39.15	P=0.5273<br>P=0.0315<br>P<0.0001	N/A	N/A
	c	Repeated measures 2-way ANOVA (cage x treatment)	Veh: 12 mice; LSD=15 mice	Interaction:F (1, 25) = 1.309<br>Treatment:F (1, 25) = 0.5644<br>Cage:F (1, 25) = 76.55	P=0.2633<br>P=0.4595<br>P<0.0001	N/A	N/A

	D	Student unpaired 2-tailed t test	Veh: 12 mice; LSD=15 mice	t=0.5064, df=25	0.617	N/A	N/A
	E	Student unpaired 2-tailed t test	Veh: 12 mice; LSD=15 mice	t=0.4827, df=25	0.6335	N/A	N/A
	F	Student unpaired 2-tailed t test	Veh: 12 mice; LSD=15 mice	t=1.002, df=25	0.326	N/A	N/A
	G	Repeated measures 2-way ANOVA (location x treatment)	Veh: 12 mice; LSD=15 mice	Interaction:F (2, 50) =1.043<br>Stranger:F (2, 50) = 76.35<br>Treatment:F(1,25)=0.3239	P=0.3599<br>P<0.0001<br>P=0.5743	N/A	N/A
	H	Repeated measures 2-way ANOVA (location x treatment)	Veh: 12 mice; LSD=15 mice	Interaction:F (2, 50) =1.043<br>Stranger:F (2, 50) = 32.08<br>Treatment:F(1,75)=1.329	P=0.3600<br>P<0.0001<br>P=0.2599	N/A	N/A
**S6**	A			t=1.213, df=18	0.2407	N/A	N/A

		Student unpaired 2-tailed t test	Veh:11 mice; LSD= 9 mice				
	B	Student unpaired 2-tailed t test	Veh:11 mice; LSD= 9 mice	t=1.101, df=18	0.2852	N/A	N/A
	C	Student unpaired 2-tailed t test	Veh:11 mice; LSD= 9 mice	t=0.008302, df=8	0.9936	N/A	N/A
	E	Student unpaired 2-tailed t test	Veh: 10 mice; LSD=9 mice	t=1.065, df=17	0.3016	N/A	NA
	F	Student unpaired 2-tailed t test	Veh: 10 mice; LSD=9 mice	t=0.6573, df=17	0.5198	N/A	NA
	G	Student unpaired 2-tailed t test	Veh: 10 mice; LSD=9 mice	t=0.2652, df=19	0.7937	N/A	NA

Table S2. Detailed statistical information.

SI References

1. D. De Gregorio *et al.*, The hallucinogen d-lysergic diethylamide (LSD) decreases dopamine firing activity through 5-HT1A, D2 and TAAR1 receptors. *Pharmacol Res* **113**, 81-91 (2016).
2. D. Marona-Lewicka, R. A. Thisted, D. E. Nichols, Distinct temporal phases in the behavioral pharmacology of LSD: dopamine D 2 receptor-mediated effects in the rat and implications for psychosis. *Psychopharmacology* **180**, 427-435 (2005).
3. F. R. Bambico, N. Katz, G. Debonnel, G. Gobbi, Cannabinoids elicit antidepressant-like behavior and activate serotonergic neurons through the medial prefrontal cortex. *Journal of Neuroscience* **27**, 11700-11711 (2007).
4. A. Aguilar-Valles *et al.*, Translational control of depression-like behavior via phosphorylation of eukaryotic translation initiation factor 4E. *Nature communications* **9**, 2459 (2018).
5. G. Gobbi *et al.*, Antidepressant-like activity and modulation of brain monoaminergic transmission by blockade of anandamide hydrolysis. *Proceedings of the National Academy of Sciences* **102**, 18620-18625 (2005).
6. G. Paxinos, K. B. Franklin, *Paxinos and Franklin's the mouse brain in stereotaxic coordinates* (Academic press, 2019).
7. F. R. Bambico *et al.*, Genetic deletion of fatty acid amide hydrolase alters emotional behavior and serotonergic transmission in the dorsal raphe, prefrontal cortex, and hippocampus. *Neuropsychopharmacology* **35**, 2083-2100 (2010).
8. F. R. Bambico, B. Lacoste, P. R. Hattan, G. Gobbi, Father absence in the monogamous California mouse impairs social behavior and modifies dopamine and glutamate synapses in the medial prefrontal cortex. *Cerebral Cortex* **25**, 1163-1175 (2013).
9. C. R. Ashby Jr, E. Edwards, R. Y. Wang, Electrophysiological evidence for a functional interaction between 5-HT1A and 5-HT2A receptors in the rat medial prefrontal cortex: An Iontophoretic study. *Synapse* **17**, 173-181 (1994).
10. M. El Mansari, P. Blier, In vivo electrophysiological characterization of 5-HT receptors in the guinea pig head of caudate nucleus and orbitofrontal cortex. *Neuropharmacology* **36**, 577-588 (1997).
11. F. R. Bambico *et al.*, A Key Role for Prefrontocortical Small Conductance Calcium-Activated Potassium Channels in Stress Adaptation and Rapid Antidepressant Response. *Cerebral Cortex* (2019).
12. A. Parsegian, W. B. Glen Jr, A. Lavin, R. E. See, Methamphetamine self-administration produces attentional set-shifting deficits and alters prefrontal cortical neurophysiology in rats. *Biological psychiatry* **69**, 253-259 (2011).
13. S. Wiebe *et al.*, Inhibitory interneurons mediate autism-associated behaviors via 4E-BP2. *Proceedings of the National Academy of Sciences* **116**, 18060-18067 (2019).
14. T. Cassano *et al.*, Evaluation of the emotional phenotype and serotonergic neurotransmission of fatty acid amide hydrolase-deficient mice. *Psychopharmacology* **214**, 465-476 (2011).
15. I. Gantois *et al.*, Metformin ameliorates core deficits in a mouse model of fragile X syndrome. *Nature medicine* **23**, 674 (2017).

16. B. D. Heifets *et al.*, Distinct neural mechanisms for the prosocial and rewarding properties of MDMA. *Science Translational Medicine* **11** (2019).
17. S. Comai, R. Ochoa-Sanchez, S. Dominguez-Lopez, F. R. Bambico, G. Gobbi, Melancholic-like behaviors and circadian neurobiological abnormalities in melatonin MT1 receptor knockout mice. *International Journal of Neuropsychopharmacology* **18** (2015).
18. M. M. Diehl *et al.*, Active avoidance requires inhibitory signaling in the rodent prelimbic prefrontal cortex. *Elife* **7**, e34657 (2018).
19. J. Mattis *et al.*, Principles for applying optogenetic tools derived from direct comparative analysis of microbial opsins. *Nature methods* **9**, 159 (2012).
20. B. D. Hare *et al.*, Optogenetic stimulation of medial prefrontal cortex Drd1 neurons produces rapid and long-lasting antidepressant effects. *Nature communications* **10**, 1-12 (2019).
21. D. J. Calu *et al.*, Optogenetic inhibition of dorsal medial prefrontal cortex attenuates stress-induced reinstatement of palatable food seeking in female rats. *Journal of Neuroscience* **33**, 214-226 (2013).
